# Sample size recalculation based on the overall success rate in a randomized test-treatment trial with restricting randomization to discordant pairs

**DOI:** 10.1186/s12874-024-02410-3

**Published:** 2025-03-18

**Authors:** Caroline Elzner, Amra Pepić, Oke Gerke, Antonia Zapf

**Affiliations:** 1 ACOMED statistik, Fockestrasse 57, Leipzig, 04275 Germany; 2 Institute of Medical Biometry and Epidemiology, University Medical Center Hamburg-Eppendorf (UKE), Christoph-Probst Weg 1, Hamburg, 20246 Germany; 3 Department of Clinical Research, Research Unit for Clinical Physiology and Nuclear Medicine, University of Southern Denmark, Campusvej 55, Odense C, 5230 Denmark

**Keywords:** Adaptive design, Sample size recalculation, Diagnostic test, Discordance design, Overall success rate

## Abstract

**Background:**

Randomized test-treatment studies are performed to evaluate the clinical effectiveness of diagnostic tests by assessing patient-relevant outcomes. The assumptions for a sample size calculation for such studies are often uncertain.

**Methods:**

An adaptive design with a blinded sample size recalculation based on the overall success rate in a randomized test-treatment trial with restricting randomization to discordant pairs is proposed and evaluated by a simulation study. The results of the adaptive design are compared to those of the fixed design.

**Results:**

The empirical type I error rate is sufficiently controlled in the adaptive design as well as in the fixed design and the estimates are unbiased. The adaptive design achieves the desired theoretical power, whereas the fixed design tends to be over- or under-powered.

**Conclusions:**

It may be advisable to consider blinded recalculation of sample size in a randomized test-treatment study with restriction of randomization to discordant pairs in order to improve the conduct of the study. However, there are a number of study-related limitations that affect the implementation of the method which need to be considered.

**Supplementary Information:**

The online version contains supplementary material available at 10.1186/s12874-024-02410-3.

## Introduction

The purpose of diagnostic testing is to gather data that will help clinicians manage their patients. When considering a diagnostic test, physicians often set a high priority on the test's diagnostic accuracy. According to the Committee for Medicinal Products for Human Use [1], it indicates the “certainty of the diagnosis” and is determined by the sensitivity, the probability of classifying a diseased individual as diseased, and specificity, the probability of classifying a non-diseased individual as non-diseased, of the diagnostic test [2]. However, the recommendation is to assess whether sufficient diagnostic accuracy is also clinically relevant by resulting in superior patient management and improved patient-relevant outcomes [3, 4]. Randomized diagnostic trials are performed to evaluate the clinical effectiveness of diagnostic tests in terms of a patient-relevant outcome, such as mortality, quality of life or progression-free survival.

### Randomized test-treatment studies

The basic structure includes the randomization of study subjects in two different test-treatment pathways, where a diagnostic test under investigation is applied. After receiving the test results, a pre-defined management strategy based on these test results is performed and finally a patient-relevant outcome is measured and evaluated. The primary objective is to assess which diagnostic test-treatment pathway implicates better patient outcome. The implication of a binary diagnostic test result can be straightforward (e.g. minimally invasive surgical procedures with clinically clearly indicated follow-up visits) or quite complicated when, for instance, the outcome of an imaging test suggests surgery. Therefore, both diagnostic testing and patient management – referred to as “test-treatment strategies” – are evaluated jointly. For the sake of simplicity, we assume throughout this article that the study subjects receive predefined treatment options, i.e., treatment I and treatment II.

Several study designs exist in the literature [5–7] of which the classical strategy design is the most common one in practice [8–11]. In the classical design, the study population is randomized into two study arms and assigned to one of two testing methods. This enables the comparison of a novel diagnostic test and an already established test.

### Motivation of this article

Randomizing all included patients in test-treatment studies tends to be inefficient in terms of sample size since a difference between the two study arms is to be expected only in the patients with a discordant test result [12]. Alternatively, the discordance design or randomized test-treatment trial with restricting randomization to discordant pairs is recommended [7, 12, 13]. Here, two testing procedures are applied to the complete study population. Then, the study subjects are randomized to one of the predetermined treatment options only if their test results from the two diagnostic tests are discordant. Patients whose test results are concordant may receive a predetermined treatment option. The primary outcome is evaluated after receiving treatment based on the test results [7]; however, the primary analysis is restricted to the discordant cases.

Calculating the sample size is a crucial step to detect a desired test-treatment effect with sufficient power while maintaining the type I error rate. Depending on the primary endpoint and study design, randomized test-treatment trials require additional information beyond type I error, desired power, and treatment effect assumptions [14]. Since entire test-treatment pathways are considered, assumptions about diagnostic accuracy (sensitivity and specificity), disease prevalence, and treatment effects in correctly and incorrectly diagnosed individuals are necessary [7]. A major challenge is to elaborate assumptions for these parameters due to limited evidence from literature or prior studies, leading to unreliable sample size calculations and over- or under-powered studies [14]. Therefore, it may be beneficial to allow sample size adjustments during an ongoing trial based on information collected up to a predefined point in time. Such modifications during an ongoing trial are one form of adaptive designs, which are common in traditional therapeutic trials but less common in diagnostic trials [15, 16]. An adaptive clinical trial design enables prospectively planned adjustments to one or more study components based on information gathered from trial participants during the ongoing trial [17, 18].

Sample size recalculation addresses uncertainties in trial planning. Hot et al. (2022) [14] assessed blinded recalculation using disease prevalence in a classical strategy design. Blinding until a predefined time point prevents the detection of differences between the study arms until then, as well as the testing of null hypotheses [19]. Nuisance parameters, not of primary interest but affecting comparisons, guide blinded recalculations. For continuous outcomes, pooled variance is used; for binary outcomes, overall success rate. Balancing trial integrity, type I error control, and blinding is crucial [20]. In their study, Hot et al. (2022) [14] compared adaptive to fixed designs, finding desired power and controlled type I error. However, varying treatment effects may lead to unrealistically large sample sizes, rendering implementation impractical. They suggested considering randomization restricted to patients with discordant test results to mitigate this issue. With this article, we aim to address this important gap by adapting the internal pilot study approach proposed by Wittes and Brittain (1990) [21] from the classical strategy design to the discordant design.

### Aim of this study

The aim of this paper is to propose and evaluate a method for blinded sample size recalculation based on the overall success rate in a randomized test-treatment trial with restriction of randomization to discordant pairs.

The adaptive design involves recalculating sample size in a blinded manner, following the internal pilot study approach proposed by Wittes and Brittain (1990) [21]. In this approach, the initial phase is termed the “pilot phase,” where nuisance parameters are estimated, and sample size is recalculated in an interim analysis. All observations are treated as originating from a single study during final data analysis. The choice of nuisance parameter depends on study design and primary endpoint; for our binary endpoint scenario, the overall success rate is selected and re-estimated during interim analysis. Thus, the adaptive design utilizes this re-estimated success rate to recalculate sample size in a blinded manner.

By means of a simulation study, we assessed whether the adaptive design achieves its operational goals, i.e.,

- the estimates are unbiased, the type I error rate is not inflated, and the actual power is equivalent to the pre-specified theoretical power;
- whether the recalculated sample sizes are realistic;
- the large difference between the theoretical and actual power in the fixed design justifies the extra effort required to use an adaptive design.

The paper is structured the following way. The Methods section includes

1. the design considerations as the basis for the sample size calculation in a randomized test-treatment trial (fixed design),
2. the description of the procedure of a blinded sample size recalculation based on the overall success rate (adaptive design),
3. an example study with
  - a hypothetical follow-up study (sample size calculation in the fixed design),
  - an application of a blinded sample size recalculation to the hypothetical follow-up study (adaptive design), and
4. a practical implementation within a simulation study.

The Results section comprises the simulation study findings in accordance with the aforementioned goals. Finally, we close with a discussion and conclusion.

## Methods

### Fixed design

In the following, we consider a discordance randomized test-treatment trial evaluating a binary patient outcome and refer to the notation in Hot et al. (2021) [7]. In general, test-treatment strategies are compared where the particular test is linked to the treatments by the test results. Firstly, let $D\in \{+,-\}$ be the true disease status of the individuals included in the trial, where $D = +$ denotes those with the target condition (i.e. the truly diseased) and $D = -$ denotes those without. Hence, $\pi = P(D = +)$ refers to the proportion with the target condition in the study. If the target condition implies the presence of a disease, then $\pi$ indicates the disease prevalence of the population.

In the first phase of the trial, two binary diagnostic tests – an experimental test $A$ and a comparator test $B$, with test $A$ performing better in regard to the diagnostic accuracy (sensitivity *Se* and specificity *Sp*) compared to test $B$ – are applied in the whole study population implying a paired study design, i.e., all subjects undergo both tests (paired design). Let ${\rm T}\in \{A, B\}$ denote the test applied to a subject and ${R}_{\rm T}\in \{+,-\}$ the result of the corresponding test ${\rm T}$. Randomization is then restricted only to a subgroup of patients with discordant test results to follow either a management strategy $M\in \left\{I, II\right\}$ based on test results of test $A$ or $B$, i.e. $M= m({R}_{\rm T} )$ with $m(+) = I$ and $m(-) = II$. The management strategy is predetermined if both tests are concordant, for example subjects with positive test results receive management strategy *I* and subjects with negative test results receive management strategy *II*.

Management strategy $I$ may be a more invasive treatment or therapeutic approach which should work better for truly diseased subjects, and management strategy $II$ may represent a standard of care which should work better in truly non-diseased subjects. Finally, after receiving a management strategy, the subject relevant binary response variable ${Y}_{T}$ is measured, restricted to subjects with discordant test results, with ${Y}_{T} \sim Bin({n}_{T}, {\theta }_{T}^{disc})$, *T* ∈ {*A*, B}, and ${n}_{T}=\frac{{n}_{disc}}{2}$ as the number of subjects with discordant test results randomized to follow the management strategy based on the test result ${R}_{T}\in \left\{+,-\right\}$ of test *T*. ${\theta }_{T}^{disc}$ denotes the expected outcome in terms of the expected proportion of favourable outcome, i.e. the single success rate based on diagnostic test *T* and subsequent corresponding management. Note that we assume a 1:1 randomization here, otherwise ${n}_{T}$ would have to be adapted accordingly to the randomization ratio.

The hypothesis of interest refers to test whether there is a difference in outcome between the management strategy based on the results of test *A* and the management strategy based on the results of test *B*, restricted to the discordant pairs. The hypotheses can be formulated in terms of the difference of the single success rates ${\Delta }_{disc}={\theta }_{A}^{disc}-{\theta }_{B}^{disc}$ as follows:

$${H}_{0}:{\Delta }_{disc}=0\,vs.\,{H}_{1}:{\Delta }_{disc}\ne 0$$

with ${\theta }_{t}^{disc}:=E({Y}_{T}|{\rm T}=t, {R}_{A}\ne {R}_{B})$ denoting the expected single success rate in each test-treatment arm based on test $T=\{A,B\}$. Subsequently, the expected outcomes for test $T=t$ can be expressed as:

1 $${\theta }_{t}^{disc}=\sum_{\begin{array}{c}{r}_{t}\in \left\{+,-\right\}, \\ d\in \{+,-\}\end{array}}\frac{\left({\mu }_{m\left({r}_{t}\right){r}_{t}d}^{t}\cdot [P\left({R}_{t}={r}_{t}|D=d\right)-P\left({R}_{A}={r}_{t},{R}_{B}={r}_{t}|D=d\right)]\right)\cdot P\left(D=d\right)}{f}$$

where ${\mu }_{m\left({r}_{t}\right){r}_{t}d}^{t}=E\left({Y}_{T}|M=m, {R}_{t}={r}_{t}, D=d\right)$ denotes the expected outcome in the respective subgroup of subjects [7], and $P\left({R}_{t}={r}_{t}|D=d\right)$, for ${r}_{t},d \in \left\{+,-\right\}$, refers to the sensitivity, false-positive rate, false-negative rate, and specificity, respectively. Further, $P\left({R}_{A}={r}_{t},{R}_{B}={r}_{t}|D=d\right)$, for ${r}_{t},d \in \left\{+,-\right\}$, refers to the true positive positive rate (TPPR), false positive positive rate (FPPR), false negative negative rate (FNNR), and the true negative negative rate (TNNR), respectively. The final term in the numerator, $P\left(D=d\right)$, represents the proportion of patients with and without the target condition, and $f=P\left({R}_{A}\ne {R}_{B}\right)=\pi \cdot {f}^{+}+\left(1-\pi \right)\cdot {f}^{-}$ denotes the overall discordant fraction that is calculated as sum of the discordant fractions for the population with ${(f}^{+})$ and without ${(f}^{-})$ the target condition weighted with the disease prevalence. The discordant fractions for the population with and without the target condition, respectively are bounded [22, 23] as follows:

2 $$\left|Se_A-Se_B\right|\leq f^+\leq Se_A+Se_B-2\cdot Se_ASe_B\;{\text{and}}\;\left|Sp_A-Sp_B\right|\leq f^-\leq Sp_A+Sp_B-2\cdot Sp_ASp_B$$

The following applies to all further considerations: $S{e}_{t}= P\left({R}_{t}=+|D=+\right)$ and $S{p}_{t}= P\left({R}_{t}=-|D=-\right)$, for $t=\left\{A,B\right\}$, denote the sensitivity and specificity of test $t$, respectively. Additionally, the TPPR, TNNR, FPPR, and FNNR are expressed as:

$$TPPR=P\left(R_A=+,R_B=+\vert D=+\right)={\frac12\cdot(}Se_A+{Se}_B-f^+),$$

$$TNNR=P\left(R_A=-,R_B=-\vert D=-\right)={\frac12\cdot(}Sp_A+{Sp}_B-f^-),$$

$$FNNR=P\left(R_A=-,R_B=-\vert D=+\right)=1-{\frac12\cdot(}Se_A+{Se}_B+f^+),$$

$$FPPR=P\left(R_A=+,R_B=+\vert D=-\right)=1-{\frac12\cdot(}Sp_A+{Sp}_B+f^-),$$

The total sample size for binomial trials depends on the success rates ${\theta }_{A}, {\theta }_{B}$, under tests $A$ and $B$, the type I error rate, $\alpha$, the power, $1-\beta$, and the discordant fraction, $f$. The sample size of discordant cases ${n}_{disc}={n}_{A}+{n}_{B}$ needed for this trial design can be calculated for the balanced design by inserting ${\theta }_{A}^{disc}$ and ${\theta }_{B}^{disc}$ in the following formula [24]:

3 $${n}_{A}={n}_{B}= {\left[\sqrt{2\overline{\theta }\left(1-\overline{\theta }\right)}{z}_{1-{}^{\alpha }\!\left/ \!{}_{2}\right.}+\sqrt{{\theta }_{A}^{disc}\left(1-{\theta }_{A}^{disc}\right)+{\theta }_{B}^{disc}\left(1-{\theta }_{B}^{disc}\right)}{z}_{1-\beta }\right]}^{2}/{{\Delta }_{disc }}^{2}$$

Here, the term

4 $$\overline{\theta }= \frac{({\theta }_{A}^{disc}+{\theta }_{B}^{disc})}{2}$$

denotes the overall success rate [24]. The total sample size is then calculated by dividing the total number of discordant cases by the overall discordant rate $f$: $N=\frac{{n}_{A}+{n}_{B}}{f}=\frac{{n}_{disc}}{f}$.

Because this article is a continuation of previous work [7], we have again formulated the methods for a binary endpoint. However, they can be easily applied to continuous endpoints. In this case, additional assumptions concerning the variation of $Y$ are needed, which is usually a nuisance parameter in the context of blinded sample size recalculation [25–28].

### Adaptive design

A randomized test-treatment trial's sample size calculation is based on critical information that may not be available in the planning phase or is subject to high uncertainty. It may be advisable to use interim study data to examine the validity of any inaccurate assumptions. To estimate a nuisance parameter, a blinded sample size recalculation involves performing an interim analysis without revealing the test-treatment assignment. In this context, the overall success rate constitutes the nuisance parameter which is used to adjust the sample size in order to preserve the power without affecting the type I error rate and prevent unblinding the study.

The following steps are considered [21]:

1. Using the sample size formula in (3) and the formula for the calculation of the success rates in (1) and (4) as well as the formula for the calculation of the discordance rates in (2), the initial total sample size ${N}_{init}=\frac{{n}_{disc}}{f}$ is calculated based on assumptions regarding the sensitivity and specificity of the two diagnostic tests $A$ and $B$, i.e. $S{e}_{A}$, $S{e}_{B}$, and $S{p}_{A}$, $S{p}_{B}$, respectively, as well as assumptions regarding the disease prevalence $\pi$ and the expected outcomes ${\mu }_{m\left({R}_{t}\right){r}_{t}d}^{t}$ with ${r}_{t}, d\in \left\{+,-\right\}$, $m\left({R}_{t}\right)\in \{I, II\}$ and $t\in \{A,B\}$.
2. Subjects are recruited until a predetermined fraction ($\varphi$) of the initial total sample size ${N}_{init}$, denoted by ${N}_{part}= {N}_{init}\cdot \varphi$, is obtained. At the interim stage of the trial, the nuisance parameter, i.e., ${\widehat{\theta }}_{est}$, the overall success rate (and optionally the overall discordant rate, which is technically possible and implemented in the provided R Code but was not investigated in this study) is estimated.
3. Substituting ${\widehat{\theta }}_{est}$ for $\overline{\theta }$ in (3) (and optionally $\widehat{f}$ for $f$) provides the recalculated sample size of discordant cases ${n}_{disc}^{adapt}$ and the recalculated total sample size ${N}_{adapt}=\frac{{n}_{disc}^{adapt}}{f}$ that the current data suggest should have been specified for the trial. If the recalculated total sample size ${N}_{adapt}$ is larger than the already recruited sample size ${N}_{part}$, further patients will be recruited until the adjusted sample size will be reached. Otherwise, no further recruitment beyond ${N}_{part}$ is necessary.
4. The study is analysed based on the unadjusted type I error level due to the blinded character of the recalculation procedure.

### An example study

To illustrate the sample size calculation in a randomized test-treatment trial with restricting randomization to discordant pairs, a multicentre, randomized, prospective trial comparing hysterosalpingo foam sonography (HyFoSy) with hysterosalpingography (HSG) as first-choice tubal patency test in infertile women, the FOAM study, is considered [13, 29].

The binary primary outcome, ongoing pregnancy leading to live birth within 12 months after inclusion, was assessed for participating women who underwent both HyFoSy and HSG in randomized order. In case of discordant test results, women were randomly allocated to either a management strategy based on HyFoSy or one based on HSG result.

The study was originally planned as a non-inferiority study, i.e., HyFoSy should not be inferior to HSG, but for simplicity it is assumed to be a superiority study, which entails some minor modifications.^1^

#### Sample size calculation (fixed design)

We assumed a disease prevalence of 20% (mean value of reported 11% and 30% in [29]), i.e., $\pi =0.2$, and a sensitivity and specificity for HyFoSy of 87% and 94%, i.e., $S{e}_{HyFoSy}= P\left({R}_{t}=+|D=+\right)=0.87$ and $S{p}_{HyFoSy}= P\left({R}_{t}=-|D=-\right)=0.94$ [30] and for HSG of 85% and 84%, i.e., ${Se}_{HSG}= P\left({R}_{t}=+|D=+\right)=0.85$ and $S{p}_{HSG}= P\left({R}_{t}=-|D=-\right)=0.84$ [31]. Therefore, the minimal discordant rate for the truly diseased and non-diseased population can be determined as follows:

$${f}_{min}^{+}=\left|{Se}_{HyFoSy}-{Se}_{HSG}\right|=\left|0.87-0.85\right|=0.02,$$

$$f_{min}^-=\left|Sp_{HyFoSy}-{Sp}_{HSG}\right|=\left|0.94-0.84\right|=0.1.$$

The overall discordant rate using the minimal discordant rates for the two populations is

$$\begin{aligned}{f}_{min}&= \pi \cdot {f}_{min}^{+}+\left(1-\pi \right)\cdot {f}_{min}^{-} \\&=0.2\cdot 0.02+0.8\cdot 0.1=0.084.\end{aligned}$$

This yields:

$$TPPR={\frac12\cdot(}Se_{HyFoSy}+{Se}_{HSG}-f_{min}^+)=\frac12\cdot\left(0.87+0.85-0.02\right)=0.85,$$

$$TNNR={\frac12\cdot(}Sp_{HyFoSy}+{Sp}_{HSG}-f_{min}^-)=\frac12\cdot\left(0.94+0.84-0.1\right)=0.84,$$

$$FNNR=1-{\frac12\cdot(}Se_{HyFoSy}+{Se}_{HSG}+f_{min}^+)=1-\frac12\cdot\left(0.87+0.85+0.02\right)=0.13$$

$$FPPR=1-{\frac12\cdot(}Sp_{HyFoSy}+{Sp}_{HSG}+f_{min}^-)=1-\frac12\cdot\left(0.94+0.84+0.1\right)=0.06.$$

Assuming that it makes no difference whether the choice of management strategy is based on HyFoSy or HSG, we assume for both groups that the chance of success in the truly diseased population is 0.2 when a diagnosis is correctly made (true positive). The chance of success in the truly non-diseased population is assumed to be 0.6 when subjects are correctly classified as non-diseased (true negative). Among subjects who are falsely diagnosed (false positive), the chance of success is assumed to be 0.5. For subjects who are falsely classified as non-diseased (false negative), the assumed chance of success is 0.1. Based on these assumed and obtained values, the single success rates are 58% for HyFoSy and 48% for HSG, resulting in an overall success rate of 53%. To test the null hypothesis of no difference between the single success rates of the two groups, we set the type I error rate at 5% and the power at 80%. By substituting the obtained values into formula (3), ${n}_{A}={n}_{B}=$ 390 subjects with discordant test results are required in each group (780 discordant cases in total and *N* = 9286 subjects overall) to detect a 10% difference in the single success rates between the two groups. It is important to note that only minimal effort is needed for concordant cases, as routine care is provided. Ideally, these cases should be included in a register to ensure structured follow-up.

#### Sample size recalculation (adaptive design)

For an adaptive design, after 50% of all intended 9286 subjects (i.e., 4643 subjects) have been recruited, a sample size recalculation is performed based on the re-estimated overall success rate. Let us consider some specific hypothetical values of the re-estimated overall success rate ${\widehat{\theta }}_{est}$, obtained in the interim analysis. Since the single success rates cannot be determined in a blinded manner during the interim analysis, the corresponding single success rates are calculated using the re-estimated overall success rate and the initially assumed delta, ${\Delta }_{disc, init}$, to ensure that the initial rate difference is maintained:

$${\widehat{\theta }}_{A}^{disc}= {\widehat{\theta }}_{est}+\frac{{\Delta }_{disc, init}}{2}$$

$${\widehat{\theta }}_{B}^{disc}= {\widehat{\theta }}_{est}-\frac{{\Delta }_{disc, init}}{2}$$

The respective adjusted (recalculated) sample size of discordant cases ${n}_{disc}^{adapt}$ as well as the adjusted total sample size ${N}_{adapt}$ are calculated by substituting the obtained success rates in formula (3).

Table 1 presents the adjusted sample sizes and the differences between the adjusted and the initially planned sample sizes for various hypothetical values of the re-estimated overall success rate:

**Table 1:** Adjusted sample sizes for various hypothetical values of the re-estimated overall success rate

**Hypothetical re-estimated overall success rate** ${\widehat{{\varvec{\theta}}}}_{{\varvec{e}}{\varvec{s}}{\varvec{t}}}$	${{\varvec{n}}}_{{\varvec{d}}{\varvec{i}}{\varvec{s}}{\varvec{c}}}^{{\varvec{a}}{\varvec{d}}{\varvec{a}}{\varvec{p}}{\varvec{t}}}$	${{\varvec{N}}}_{{\varvec{a}}{\varvec{d}}{\varvec{a}}{\varvec{p}}{\varvec{t}}}$	**Absolute difference:** ${{\varvec{n}}}_{{\varvec{d}}{\varvec{i}}{\varvec{s}}{\varvec{c}}}^{{\varvec{a}}{\varvec{d}}{\varvec{a}}{\varvec{p}}{\varvec{t}}}-\boldsymbol{ }{{\varvec{n}}}_{{\varvec{d}}{\varvec{i}}{\varvec{s}}{\varvec{c}}}^{{\varvec{i}}{\varvec{n}}{\varvec{i}}{\varvec{t}}}$	**Relative difference:** $\frac{{{\varvec{n}}}_{{\varvec{d}}{\varvec{i}}{\varvec{s}}{\varvec{c}}}^{{\varvec{a}}{\varvec{d}}{\varvec{a}}{\varvec{p}}{\varvec{t}}}-\boldsymbol{ }{{\varvec{n}}}_{{\varvec{d}}{\varvec{i}}{\varvec{s}}{\varvec{c}}}^{{\varvec{i}}{\varvec{n}}{\varvec{i}}{\varvec{t}}}}{{{\varvec{n}}}_{{\varvec{d}}{\varvec{i}}{\varvec{s}}{\varvec{c}}}^{{\varvec{i}}{\varvec{n}}{\varvec{i}}{\varvec{t}}}}$
0.35	712	8478	-68	-0.0872
0.45	776	9240	-4	-0.0051
${\overline{\theta }}_{init}=0.53$	${n}_{init}^{disc}=780$	${N}_{init}=9286$		
0.60	752	8954	-28	-0.0359
0.70	658	7834	-122	-0.1564

It can be noted that there is a small sample size reduction in the adaptive design compared to the fixed design, but this reduction may only be relevant in the case of large differences between the initially assumed and the hypothetical re-estimated overall success rate.

It might be reasonable that the overall success rate, and thus the corresponding variance, does not much influence the sample size. This can be illustrated through an exemplary sample size calculation for testing two proportions using the Z-test, conducted with PASS 16.0.3 (see Supplemental Table 1, Additional File 1). Notably, with a single success rate (P2) ranging from 0.3 to 0.5 and an absolute delta (D1) between 0.15 and 0.3, there is little variation in the sample size. Only for a delta (D1) of 0.1, there is a bit of variation in sample size depending on the single success rates (see Supplemental Fig. 1, Additional File 1). When the overall success rate diverges significantly from 0.5, the corresponding reduction in variance allows for greater precision in estimating the parameters of interest. Consequently, with lower variance, the required sample size to maintain a specific level of statistical power can also be reduced.

 In 2008, Gerke et al. [32] described the unexpected finding that statistical power increases with greater agreement between tests. As the success rate diverges significantly from 0.5, it can be hypothesized that the level of agreement between the two tests correspondingly increases.

### Simulation study

In the simulation study, we used parameter values derived from the example study. This simulation aimed to evaluate the efficacy of the sample size recalculation method within the framework of a randomized test-treatment trial with randomization restricted to discordant pairs. The investigation involved exploring various data scenarios.

The simulation study was conducted and documented in a structured manner as suggested by Burton et al. [33] and Morris et al. [34].

For each scenario, we consider the targets of the analysis: bias, type I error and the actual power of the adaptive design, in order to examine whether the adaptive design does not inflate the type I error rate and achieves the pre-specified power.

The findings of the adaptive design will be compared with those of the fixed design, which does not include an adaptive component. The simulation study will provide answers to the following questions:

- Are the estimated parameters unbiased?
- Is the type I error rate sufficiently controlled?
- Does the empirical power equal the predetermined target power of 80%?
- Does the difference in power between the two designs warrant the extra effort required to use an adaptive design?

The empirical type I error rate is calculated as proportion of *p*-values from testing the null hypothesis of no difference in each simulated sample that are below the $5{\%}$ significance level when the null hypothesis is true. The power is determined as the proportion of simulation samples in which the null hypothesis of no effect is rejected at the two-sided $5{\%}$ significance level when the null hypothesis is false. The bias of the estimated overall success rate in the interim analysis is calculated as percentage of the true value, i.e. $\left(\frac{\left(\frac{1}{{n}^{sim}}\cdot \sum_{i=1}^{{n}^{sim}}{\widehat{\theta }}_{est,i}-{\overline{\theta }}_{true}\right)}{{\overline{\theta }}_{true}}\right)\cdot 100\%$ [33] to verify an unbiased estimation of the overall success rate in the interim analysis.

In total, 288 scenarios were simulated, i.e., three sets of the difference between the true and the initially assumed overall success rate (i.e., the pre-specified shift of $\omega \in \left\{0.05, 0.10, 0.15\right\}$), three sets of the prevalence ($\pi \in \left\{0.1, 0.2, 0.3\right\}$),^2^ two sets each from the sensitivity and specificity of test *A* ($S{e}_{A} \in \left\{0.8, 0.9\right\}, S{p}_{A} \in \left\{0.8, 0.9\right\}$) as well as test *B* under the null

($S{e}_{A}=S{e}_{B}, S{p}_{A}=S{p}_{B}$) and alternative hypothesis

$$\begin{aligned}\left((1)Se_B\right.&=Se_A-0.1,Sp_B=Sp_A-0.1;\\{(2)}Se_B&\left.=Se_A-0.2,Sp_B=Sp_A-0.2\right),\end{aligned}$$

respectively, and two sets of the discordant rate (minimal and mean). The variations of the parameters (i.e., data generation scenarios) are also given in Supplemental Table 2 (see Additional File 1). Per scenario, 10,000 replications (i.e., number of simulations) were performed.

With the purpose of varying the true overall success rate, a pre-specified shift of $\omega$ between the true and initially assumed overall success rate was introduced, assuming that the true overall success rate is greater by $\omega$ than the initially assumed overall success rate. Consequently, the true overall success rate ${\overline{\theta }}_{true}$ is larger by $\omega /2$ and the initially assumed overall success rate ${\overline{\theta }}_{init}$ is smaller by $\omega /2$ than the overall success rate in the generated data ${\overline{\theta }}_{data}$*.* This means that the data were generated using the above parameters (see Supplemental Table 2, Additional File 1) with fixed values, i.e., the true values. Then, the initially assumed single success rates were calculated using the same parameters with the same values (i.e., the true values), but corrected for the pre-specified shift by subtracting $\omega /2$ before calculating the initially assumed overall success rate and determining the initial sample size. The true overall success rate was then calculated by adding the pre-specified shift $\omega$ to the initially assumed overall success rate. The true single success rates were subsequently determined using the true overall success rate and the initially assumed delta to ensure that the initial difference in rates is maintained. The true sample size was then determined by using the obtained true success rates. The success rates determined from the generated data also had to be corrected for this pre-specified shift by adding $\omega /2$ to the determined success rates.

In order to limit the complexity of the simulation study, we assumed that the expected outcomes ${\mu }_{m\left({R}_{t}\right){r}_{t}d}^{t}$ with ${r}_{t}, d\in \left\{+,-\right\}$, $m\left({R}_{t}\right)\in \{I, II\}$ and $t\in \{A,B\}$ in the test-treatment arms were independent of the applied tests, i.e. the effect of a correct diagnosis (and consequently correct treatment) as well as a false diagnosis was assumed to be the same in both diagnostic groups, respectively. Thus, we only considered the expected outcome of management strategies $I$ and $II$ in dependence of the true disease state of the patient, i.e. ${\mu }_{md}$ with $d\in \left\{+,-\right\}, m\in \{I, II\}$.

Given that increasing effects are favourable, as demonstrated in our example study (where there is an increased chance of ongoing pregnancy leading to live birth within 12 months after inclusion), we assumed that management strategy $I$ would induce a slightly positive/curative effect in the diseased population. Therefore, the expected chance of success rate in this group is considered to be moderate, i.e. ${\mu }_{I+}=0.2$. Similarly, if non-diseased individuals receive the management strategy that is optimal for them, i.e., management strategy $II$, a relatively high chance of success rate can be expected, i.e. ${\mu }_{II-}=0.6$. Patients who tested falsely positive underwent unnecessarily an invasive treatment, leading to potential complications or side effects, resulting in a lower chance of success, i.e. ${\mu }_{I-}=0.5$. Patients who tested falsely negative and actually had the disease did not receive the necessary treatment, which may lead to disease-related complications and consequences, resulting in a lower chance of success, i.e. ${\mu }_{II+}=0.1$. In addition, in the simulation study it was defined that under the null and alternative hypothesis, apart from the overall success rate, the values for the diagnostic accuracy parameters as well as the prevalence were assumed to be correctly specified in the sample size recalculation. This assumption seems justified, as the group difference should remain the same if incorrect assumptions lead to the success rates changing equally in both groups, and the sample size is recalculated based on the overall success rate. However, if incorrect assumptions affect the success rates of the two groups differently, this would violate the basic assumption of a stable group difference in the adaptive design with blinded sample size re-estimation. Statistical significance was assessed using the two-sided Wald confidence interval for binomial proportions [35, 36]. The simulation was performed using R (Software Version 4.2.2) [37]. Additional File 2 comprises both the simulation code and a list of applied R packages.

## Results

### Type I error

**Fig. 1:**
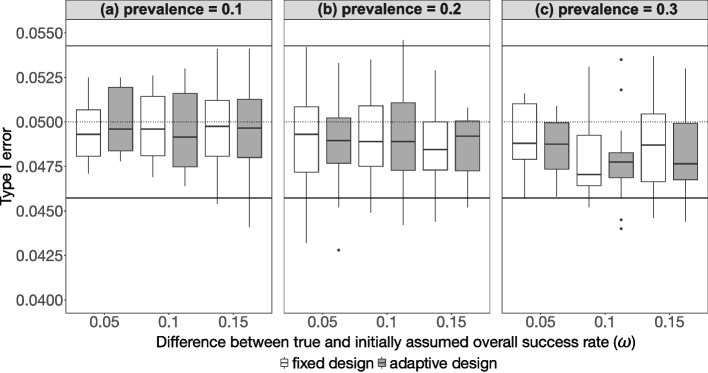
Results for the empirical type I error vs. the difference between true and initially assumed overall success rate for the 144 scenarios ($\pi \epsilon \left\{0.1, 0.2, 0.3\right\}, \omega \epsilon \left\{0.05, 0.1, 0.15\right\}, S{e}_{A}, S{p}_{A} \epsilon \left\{0.8, 0.9\right\}, S{e}_{B}, S{p}_{B} \epsilon \left\{0.6, 0.7, 0.8\right\}$) using minimal discordant rate, stratified by the prevalence. The empirical type I error rates for the fixed design and the adaptive design containing a re-estimation of the overall success rate are compared to each other. The black dotted line marks the desired theoretical type I error rate of 5% and the black solid lines mark the respective 95% prediction interval based on the Monte Carlo standard error in the simulation

Figure 1 illustrates the results of the empirical type I error rate for the adaptive and the fixed designs for 144 simulated scenarios using the minimal discordant rate. The results using the mean discordant rate are presented in Supplemental Fig. 2 [Note: There are 63 scenarios that are not included in the presentation for mean discordant rate due to data generation problems (for further details see Supplemental Table 3)]. It reveals that for most scenarios, the empirical type I error rates for the adaptive design are within the limits of the 95% prediction interval based on the Monte Carlo standard error in the simulation with the median values are mostly below the theoretical type I error rate of 5%. Only about 8% (11/144) of the scenarios using minimal discordant rate are outside the 95% prediction interval, whereby only 1 out of 144 (0.7%) scenarios is above the upper limit of the 95% prediction interval, relating to the adaptive design (scenario with prevalence = 0.2 and $\omega=0.1:Se_A=0.9,\;Sp_A=0.8,\;Se_B=0.8,\;Sp_B=0.6)$.

There are no scenarios (0/81) using mean discordant rate above the upper limit of the 95% prediction interval (see Supplemental Fig. 2). About 22% (18/81) of the scenarios are below the lower limit of the 95% prediction interval, which could be due to the properties of the 63 missing scenarios.

The maximum empirical type I error rates for both designs considering all 225 scenarios (minimal and mean discordant rate) are slightly above the theoretical type I error rate of 5%, however the corresponding median values are below this threshold as illustrated in Fig. 1. Regarding the median empirical type I error rates, the lower the prevalence, the higher the empirical type I error rates.

Additionally, there are hardly any differences regarding the empirical type I error rate among the considered differences between the true and the initially assumed overall success rate.

Hence, the type I error is neither influenced by the underestimation of the true overall success rate (Fig. 1) nor by the difference in sensitivity or specificity for test $A$ and $B$ (see Supplemental Fig. 3 and Supplemental Fig. 4).

### Power

**Fig. 2:**
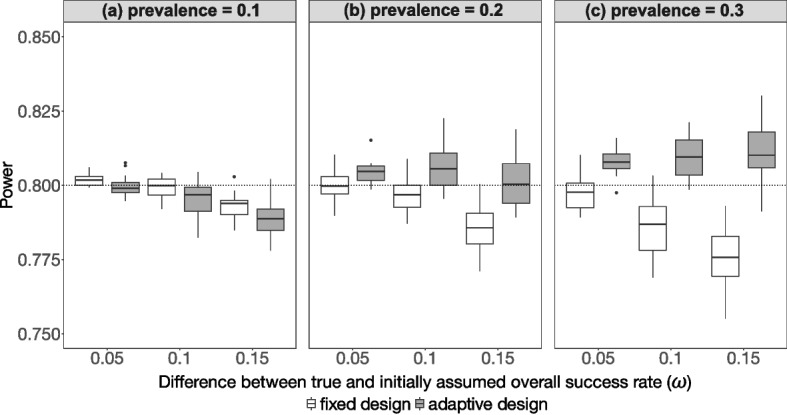
Results for the empirical power vs. the difference between true and initially assumed overall success rate for the 144 scenarios ($\pi \epsilon \left\{0.1, 0.2, 0.3\right\}, \omega \epsilon \left\{0.05, 0.1, 0.15\right\}, S{e}_{A}, S{p}_{A} \epsilon \left\{0.8, 0.9\right\}, S{e}_{B}, S{p}_{B} \epsilon \left\{0.6, 0.7, 0.8\right\}$) using minimal discordant rate, stratified by the prevalence. The empirical power in the fixed design is compared to the adaptive design containing a re-estimation of the overall success rate. The black dotted line marks the desired theoretical power of 80%

In Fig. 2, the results of the simulation study reveal that the empirical power in the adaptive design is generally close to the desired theoretical power of 80%. The effect of an initially incorrect assumption of the overall success rate is mitigated by re-estimating the overall success rate during the interim analysis. If the true overall success rate is underestimated, the empirical power in the fixed design is slightly underestimated as well. To increase the power in this situation, more subjects than initially planned have to be recruited (Fig. 2b, c).

The empirical power is also slightly affected by the assumed differences in sensitivity and specificity of test $A$ and $B$ (see Supplemental Fig. 5 and Fig. 6). In scenarios with greater difference in sensitivity between the two tests, the empirical power in the fixed design slightly decreases. This effect is reversed for the difference in specificity between the two tests, because the smaller the difference, the smaller the empirical power in the fixed design.

The effects become more pronounced with increasing disease prevalence, and the observed empirical power is nearly independent of whether the minimal (Fig. 2) or mean discordant rate (see Supplemental Fig. 7) is used. For prevalence = 0.1 (see Fig. 2a), the empirical power in the adaptive design is slightly lower than the fixed design. If the true overall success rate is underestimated by 0.15, then the empirical power in both designs is lower than the theoretical power of 80%. For prevalence = 0.2 and prevalence = 0.3 (see Fig. 2b, c), the empirical power in the adaptive design is properly controlled, while in the fixed design, the empirical power decreases to some extent, depending on the magnitude of the difference between the true and initially assumed overall success rate.

### Sample sizes

**Fig. 3:**
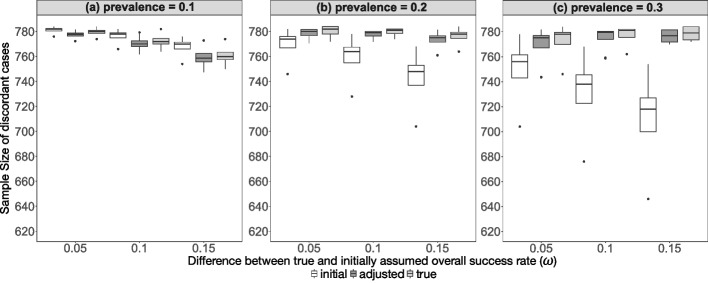
Results of the calculated initial, adjusted and true sample sizes of discordant cases vs. the difference between true and initially assumed overall success rate for the 144 scenarios ($\pi \epsilon \left\{0.1, 0.2, 0.3\right\}, \omega \epsilon \left\{0.05, 0.1, 0.15\right\}, S{e}_{A}, S{p}_{A} \epsilon \left\{0.8, 0.9\right\}, S{e}_{B}, S{p}_{B} \epsilon \left\{0.6, 0.7, 0.8\right\}$) using minimal discordant rate (based on data generated under the null hypothesis), stratified by the prevalence

For all simulated scenarios the adjusted (recalculated) sample sizes of discordant cases tended to approach the true necessary sample sizes of discordant cases, regardless of the deviations in true overall success rates (Fig. 3: using minimal discordant rate, Supplemental Fig. 8: using mean discordant rate), sensitivity (Supplemental Figs. 9, 10) or specificity (Supplemental Figs. 11, 12).

Figure 3 presents the sample sizes of discordant cases using the minimal discordant rate (based on data generated under the null hypothesis).^3^

In general, the sample sizes of discordant cases calculated using the mean discordant rate (see Supplemental Fig. 8) are on average about three times larger than using the minimal discordant rate (Fig. 3).^4^

The adjusted sample size of discordant cases is slightly larger than the initially planned sample size of discordant cases, at least for prevalence > 0.1 (Fig. 3). For a disease prevalence of 0.1, some subjects can be saved in the adaptive design, though the associated empirical power is slightly lower compared to the fixed design (Fig. 2).

For the minimal discordant rate, the absolute difference between the adjusted and the initial sample sizes of discordant cases ranges from -29 to 125 subjects, with a median difference of 15 subjects, depending on prevalence and deviations in true overall success rates, sensitivity, or specificity. This indicates that across all simulated scenarios using the minimal discordant rate, up to 29 subjects can be saved (prev = 0.1 and $\omega =0.15$), while up to 125 additional subjects may need to be recruited (prev = 0.3 and $\omega =0.15$) in the adaptive design (Fig. 3, Supplemental Table 4). For the total sample size, up to 152 subjects can be saved (prev = 0.1 and $\omega =0.15$), and up to 957 additional subjects may need to be recruited (prev = 0.3 and $\omega =0.15$) (Supplemental Table 5).

The distribution of the true necessary, initial, and recalculated total sample sizes across all scenarios is shown in Supplemental Fig. 13 and Supplemental Fig. 14.

Supplemental Fig. 15 (Supplemental Fig. 16) gives a more detailed look at the type I error rate, power and sample size of discordant cases for one specific scenario using the minimal (mean) discordant rate.

### Bias

The overall success rate is re-estimated almost without bias in the interim analysis, regardless of the deviations in true overall success rates (median percentage bias of -0.002% for data generated under the null hypothesis and median percentage bias of -0.001% for data generated under the alternative hypothesis, see Supplemental Fig. 17 and Supplemental Fig. 18), sensitivity (results not shown) or specificity (results not shown).

The overall discordant rate is re-estimated almost unbiased in the interim analysis, regardless of the deviations in true overall success rates (median percentage bias of -0.05% for data generated under the null hypothesis and median percentage bias of 0.05% for data generated under the alternative hypothesis), sensitivity (results not shown) or specificity (results not shown). There is a slight downward trend for data generated under the null hypothesis using the minimal discordant rate (median percentage bias of -0.244%, see Supplemental Fig. 19) and a slight upwards trend for data generated under the alternative hypothesis using the mean discordant rate (median percentage bias of 0.205%, see Supplemental Fig. 20).

Regardless of differences between the true and initially assumed overall success rate, sensitivity (results not shown) or specificity (results not shown) between test A and B, there is almost no bias regarding delta (i.e., the difference between the single success rates) in the adaptive design (median absolute bias of -0.000008, see Supplemental Fig. 21).

### Additional simulation

Following a reviewer’s suggestion, we also investigated additional scenarios with prevalence values ranging from 0.4 to 0.9 and ω values from -0.5 to + 0.5, keeping the other parameters unchanged (see Supplemental Table 2). However, these simulations yielded results comparable to previous findings and did not provide any relevant new insights regarding type I error, power, or the sample sizes of discordant cases. For illustrative purposes, results showing the empirical power versus the difference between the true and initially assumed overall success rate for prevalences of 0.1 and 0.5 have been added to Additional File 1 (Supplemental Fig. 22), along with a figure showing sample sizes of discordant cases versus the difference between the true and initially assumed overall success rate for these prevalences (Supplemental Fig. 23).

The additional simulation was performed using R (Software Version 4.4.1) [37].

## Discussion

This paper is a continuation of the work of Hot et al. (2022) [14], in which a simulation study was conducted to evaluate a prevalence-based sample size recalculation in a classical strategy design of a randomized test-treatment trial. A limitation of the classical design is the unpaired structure in which subjects are randomly assigned to one of two test-treatment pathways and the notion of discordant cases is not applicable. Sample sizes are usually very large, making the design very inefficient. To overcome this limitation, we investigated the extent to which a blinded adaptive design, in the form of sample size recalculation, can be integrated within a randomized test-treatment trial with restricting randomization to discordant pairs and assessed the feasibility of this design. We performed a simulation study to examine the sample size recalculation based on the re-estimated overall success rate (and optionally the overall discordant rate, which is technically possible and implemented in the provided R Code but was not investigated in this study) in case of a binary endpoint in an interim analysis.

The results of the simulation study demonstrate that the interim analysis estimates of the overall success rate, the overall discordant rate and the absolute delta are almost unbiased. The empirical type I error rate is controlled in the adaptive as well as in the fixed design across all simulated samples. The distribution of the empirical type I error rate is neither influenced by the underestimation of the true overall success rate nor by the difference in sensitivity or specificity for test $A$ and $B$.

The empirical power in the adaptive design approaches the desired theoretical power of 80% assuming that all other nuisance parameters have been correctly specified. The impact of an incorrectly assumed overall success rate is mitigated by re-estimating the overall success rate during the interim analysis. Studies using a fixed design tend to be over- or under-powered. The adjusted (recalculated) sample size of discordant cases tends to approach the true necessary sample size of discordant cases, regardless of the deviations in true overall success rates, sensitivity or specificity. Recalculating the sample size of discordant cases in an interim analysis corrects a wrongly underestimated true overall success rate and its impact on the initial sample size of discordant cases. However, it should be noted that this simulation study does not represent all possible parameter combinations.

To determine whether the gap between the power of the two designs is substantial enough to justify the additional efforts associated with using an adaptive design, we must define what constitutes a substantial gap from both logistical and practical perspectives. In our simulation study, this gap was influenced by disease prevalence as well as the magnitude of underestimation of the true overall success rate and deviations in sensitivity or specificity between tests $A$ and $B$. Additionally, it is important to consider the benefit of recalculating the sample size during the interim analysis by examining how much the recalculated sample size of discordant cases deviates from both the initial and the true necessary sample size of discordant cases. The clinical relevance and practical impact of this gain should be evaluated in terms of its implications for subjects, including outcomes, exposure to interventions, and the associated costs and efforts of implementing an adaptive design. We encourage the reader to use our R Code (provided in the Supplementary Material Additional File 2) to simulate deviations from the expected scenario by varying the respective parameters.

This study design and the associated sample size planning are inherently complex, and the adaptive aspect adds to this complexity. Therefore, we have made assumptions at various points in this paper to facilitate understanding. However, the methodological approach is generally applicable.

Nonetheless, the investigated design has some limitations.

One limitation of our simulation study is the choice of varying parameters during the data generation process and sample size calculation. In particular, the choice of expected outcomes may be unreasonable, as these values are challenging to determine in practice due to the lack of appropriate RCTs. Therefore, for simplicity, we assumed that the expected outcomes for management strategies $I$ and $II$ are fixed and equivalent. Additionally, the predefined shift value between the true and initially assumed overall success rate may be questionable. Furthermore, the chosen approach to vary “the truth” involved modifying the success rates derived from the generated data by subtracting or adding the pre-specified shift ω (to maintain the shift to the true values). However, this method may not be optimal. Typically, one or more parameters used for generating the data would be adjusted to produce the pre-specified shift ω, which would then be automatically reflected in the data. We attempted to generate the data by varying a specific parameter of the expected outcomes, but this resulted in difficulties, as the initial difference in rates was not maintained in the data generated under the alternative hypothesis. This suggests that the assumption of fixed and equivalent expected outcomes for management strategies $I$ and $II$ may not have been ideal. In future studies, one or two parameters should be identified that can be varied in the data generation process by setting the formula for the single success rate for test- $A$ based strategy equal to that of the test- $B$ based strategy.

In general, we recommend the paired design only if it is ethically acceptable and feasible to perform both tests under investigation on each study subject, the specific test results are available in a similar time interval and one test does not influence the performance of the other. Furthermore, when implementing an adaptive design with an unblinded sample size recalculation, the speed of the recruitment in relation to the time window for the assessment of the primary outcome is essential. In case where there is a quite long time period before the primary outcome can be assessed, the entire study schedule may be delayed due to insufficient data for a pre-planned interim analysis, or recruitment may be completed before adequate data have been collected. In the example study, i.e., the FOAM study, the primary outcome is assessed 12 months after inclusion in the study. To recalculate the sample size during an interim analysis, recruitment may be stopped after, for example, half of the initially planned sample size has been reached. The overall success rate needs to be re-estimated after all outcome data for the subjects included in the interim analysis have been collected. This process could take several months, resulting in significant delays to the overall study schedule. In such cases, recruitment should continue and the adaptive design cannot be recommended.

The required sample size for the paired design using the minimal discordant rate was between 3500 and 7800 subjects in total, based on 650 to 780 necessary subjects with discordant test results. The required sample sizes for these randomized test-treatment studies are quite high, so studies that include only subjects with discordant test results may be more feasible as only minimal effort is required for concordant cases.

In summary, both diagnostic tests need to be applied to all subjects, but a follow-up is not necessary for subjects with concordant test results as these subjects could be released from the study and, for instance, referred to registry studies. Only subjects with discordant test result need to be followed-up for estimating the primary outcome - making the paired design more feasible in real-world studies than the classical design.

## Conclusion

It may be advisable to consider a blinded recalculation of the sample size at the planning stage of the study to increase the chances of success and to improve the conduct of the study. Nevertheless, there are several study-related limitations that affect the method's implementation, including practicality, the required sample sizes, and meeting prerequisites.

## Supplementary Information

Supplementary Material 1: Tables and Figures.

Supplementary Material 2: Listing of results regarding empirical type I error rates as well as empirical power and R Code.

Supplementary Material 3. Listing: Empirical Type I error rate

Supplementary Material 4. Listing: Empirical Power

Supplementary Material 5. R code

## Data Availability

All data used in this study are simulated and analysed as part of the simulation study. All analyses and datasets supporting the conclusions of this article are carried out with R version 4.2.2 (2023-03-30) [37]. The R simulation code, the listing of the empirical type I error rates and the empirical power for the considered scenarios are provided in the Supplementary Material Additional File 2.

## Abbreviations

HSG: Hysterosalpingography
HyFoSy: Hysterosalpingo Foam Sonography
RCT: Randomized Controlled Trial
TNNR: True negative negative rate
TPPR: True positive positive rate
FNNR: False negative negative rate
FPPR: False positive positive rate

## Footnotes

1 In addition to the necessary modifications due to the change to a superiority study, van Rijswijk et al. used z_1- α_ = 1.64 instead of z_1- α/2_ = 1.96. The latter is generally recommended for two-sided tests according to EMA ICH E9 (1998, Chapter 5.5) [20].

2 Following a reviewer’s suggestion, we investigated additional scenarios with prevalence values ranging from 0.4 to 0.9 and ω values from -0.5 to + 0.5 (see Section " Additional simulation").

3 Using the minimal discordant rate, no difference in sample sizes of discordant cases is observed between data generated under the null and under the alternative hypothesis; hence, the results are presented only once.

4 This is not surprising as “a smaller discordant rate is associated with a bigger difference to be detected, which, in turn, demands less sample.” [12] (Page 11).
