# Supplementary material for: Sample size recalculation based on the overall success rate in a randomized test-treatment trial with restricting randomization to discordant pairs
Source: BMC Med Res Methodol. 2025 Mar 18;25:74. doi: 10.1186/s12874-024-02410-3 (PMC11921670; doi:10.1186/s12874-024-02410-3)
Supplement: Supplementary file 1 — Supplementary Material 1: Tables and Figures. [file 12874_2024_2410_MOESM1_ESM.pdf]

Additional File 1: Supplementary Material

Supplemental Table 1: Sample size calculation for the test of two proportions using the Z-test with Pooled Variance (using PASS 16.0.3): total sample size (N), sample size for each arm (N1, N2), single success rates (P1, P2), the absolute difference between the single success rates, i.e. the absolute delta (D1) [**bold** values represent no big changes in sample size (N)]

Target Power	Actual Power*	N1	N2	N	P1	P2	D1	Alpha
0.80	0.80007	199	199	398	0.2000	0.1000	0.1000	0.0500
0.80	0.80114	294	294	588	0.3000	0.2000	0.1000	0.0500
0.80	0.80006	356	356	**712**	0.4000	**0.3000**	**0.1000**	0.0500
0.80	0.80067	388	388	**776**	0.5000	**0.4000**	**0.1000**	0.0500
0.80	0.80067	388	388	**776**	0.6000	**0.5000**	**0.1000**	0.0500
0.80	0.80183	100	100	200	0.2500	0.1000	0.1500	0.0500
0.80	0.80025	138	138	276	0.3500	0.2000	0.1500	0.0500
0.80	0.80162	163	163	**326**	0.4500	**0.3000**	**0.1500**	0.0500
0.80	0.80046	173	173	**346**	0.5500	**0.4000**	**0.1500**	0.0500
0.80	0.80160	170	170	**340**	0.6500	**0.5000**	**0.1500**	0.0500
0.80	0.80260	62	62	124	0.3000	0.1000	0.2000	0.0500
0.80	0.80378	82	82	164	0.4000	0.2000	0.2000	0.0500
0.80	0.80001	93	93	**186**	0.5000	**0.3000**	**0.2000**	0.0500
0.80	0.80031	97	97	**194**	0.6000	**0.4000**	**0.2000**	0.0500
0.80	0.80001	93	93	**186**	0.7000	**0.5000**	**0.2000**	0.0500
0.80	0.80379	43	43	86	0.3500	0.1000	0.2500	0.0500
0.80	0.80071	54	54	108	0.4500	0.2000	0.2500	0.0500
0.80	0.80537	61	61	**122**	0.5500	**0.3000**	**0.2500**	0.0500
0.80	0.80362	62	62	**124**	0.6500	**0.4000**	**0.2500**	0.0500
0.80	0.80226	58	58	**116**	0.7500	**0.5000**	**0.2500**	0.0500
0.80	0.80644	32	32	64	0.4000	0.1000	0.3000	0.0500
0.80	0.80543	39	39	78	0.5000	0.2000	0.3000	0.0500
0.80	0.80029	42	42	**84**	0.6000	**0.3000**	**0.3000**	0.0500
0.80	0.80029	42	42	**84**	0.7000	**0.4000**	**0.3000**	0.0500
0.80	0.80543	39	39	**78**	0.8000	**0.5000**	**0.3000**	0.0500

* Power was computed using the normal approximation method.

Supplemental Table 2: Scenarios of the simulation study

**Parameters**	**Scenarios of the simulation study**
Disease prevalence π	$0.1, 0.2, 0.3$
Sensitivity Se_A	$0.8, 0.9$
Specificity Sp_A	$0.8, 0.9$
Discordant rate f	f_{min}, f_{mean}
Expected outcomes [only discordant cases]:	
μ_{I-}^{disc} (FP: management strategy I in truly non-diseased population)	0.5
μ_{I+}^{disc} (TP: management strategy I in truly diseased population)	0.2
μ_{II-}^{disc} (TN: management strategy II in truly non-diseased population)	0.6
μ_{II+}^{disc} (FN: management strategy II in truly diseased population)	0.1
True overall success rate $\bar{\theta}_{true} = \bar{\theta}_{init} + \omega$	$\bar{\theta}_{init} + 0.05, \bar{\theta}_{init} + 0.1, \bar{\theta}_{init} + 0.15$
Under H_0: $\Delta_{disc} = \theta_A^{disc} - \theta_B^{disc} = 0$	
Theoretical significance level α	0.05 (two-sided)
Se_B	Se_A
Sp_B	Sp_A
Under H_1: $\Delta_{disc} = \theta_A^{disc} - \theta_B^{disc} \neq 0$	
Theoretical power $1 - \beta$	0.8
Se_B, Sp_B	(1) $Se_A - 0.1, Sp_A - 0.1$
	(2) $Se_A - 0.2, Sp_A - 0.2$

Scenarios not working with the function *alpha.sc2*

There were 63 scenarios that could not be calculated with the function alpha.sc2 (for the complete R Code see the Supplementary Material Additional File 2). A detailed list of the respective scenarios is provided in Supplemental Table 3. In particular, data generation under the null hypothesis with the function *sim.parallel* did not work using following parameters:

- Mean discordant rates: $f_{mean}^{+}, f_{mean}^{-}, f_{mean}$
- Disease prevalence: $\pi = (0.1, 0.2, 0.3)$
- $\omega = (0.05, 0.1, 0.15)$
- $Se_A = Se_B = 0.8,\ Sp_A = Sp_B = 0.9$
- $Se_A = Se_B = 0.9,\ Sp_A = Sp_B = 0.8$
- $Se_A = Se_B = 0.9,\ Sp_A = Sp_B = 0.9$

R stopped the calculation of *sim.parallel* with the Error:

> *"Error in Element (1 , 2): Admissible values are in [0.8 , 0.9].*
> *Error in commonprob2sigma(commonprob, simulvals) : Matrix commonprob not admissible."*

When generating the data for the truly diseased population, the following condition must be fulfilled for the off-diagonal elements of the matrix *commonprob*, i.e. $TPPR$:

$$\max\left(0, (Se_A + Se_B - 1)\right) \leq\ TPPR \leq \min\left(Se_A, Se_B\right)$$

When generating the data for the truly non-diseased population, the following condition must be fulfilled for the off-diagonal elements of the matrix *commonprob*, i.e. $TNNR$:

$$\max\left(0, (Sp_A + Sp_B - 1)\right) \leq\ TNNR \leq \min\left(Sp_A, Sp_B\right)$$

After recalculation of $TPPR$ and $TNNR$ for the above scenarios, it became clear that either $TPPR$ or $TNNR$ or both, $TPPR$ and $TNNR$, were not within the respective desired interval. Therefore, the data generation did not work and without data the whole workflow of the simulation study collapsed.

Supplemental Table 3: Scenarios not working with the function alpha.sc2

No.	π	Se_A	Sp_A	Se_B	Sp_B	ω
1	0.1	0.8	0.9	0.7	0.7	0.05
2	0.1	0.8	0.9	0.6	0.7	0.05
3	0.1	0.9	0.8	0.7	0.7	0.05
4	0.1	0.9	0.8	0.7	0.6	0.05
5	0.1	0.9	0.9	0.8	0.7	0.05
6	0.1	0.9	0.9	0.7	0.8	0.05
7	0.1	0.9	0.9	0.7	0.7	0.05
8	0.1	0.8	0.9	0.7	0.7	0.1
9	0.1	0.8	0.9	0.6	0.7	0.1
10	0.1	0.9	0.8	0.7	0.7	0.1
11	0.1	0.9	0.8	0.7	0.6	0.1
12	0.1	0.9	0.9	0.8	0.7	0.1

No.	π	Se_A	Sp_A	Se_B	Sp_B	ω
13	0.1	0.9	0.9	0.7	0.8	0.1
14	0.1	0.9	0.9	0.7	0.7	0.1
15	0.1	0.8	0.9	0.7	0.7	0.15
16	0.1	0.8	0.9	0.6	0.7	0.15
17	0.1	0.9	0.8	0.7	0.7	0.15
18	0.1	0.9	0.8	0.7	0.6	0.15
19	0.1	0.9	0.9	0.8	0.7	0.15
20	0.1	0.9	0.9	0.7	0.8	0.15
21	0.1	0.9	0.9	0.7	0.7	0.15
22	0.2	0.8	0.9	0.7	0.7	0.05
23	0.2	0.8	0.9	0.6	0.7	0.05
24	0.2	0.9	0.8	0.7	0.7	0.05
25	0.2	0.9	0.8	0.7	0.6	0.05
26	0.2	0.9	0.9	0.8	0.7	0.05
27	0.2	0.9	0.9	0.7	0.8	0.05
28	0.2	0.9	0.9	0.7	0.7	0.05
29	0.2	0.8	0.9	0.7	0.7	0.1
30	0.2	0.8	0.9	0.6	0.7	0.1
31	0.2	0.9	0.8	0.7	0.7	0.1
32	0.2	0.9	0.8	0.7	0.6	0.1
33	0.2	0.9	0.9	0.8	0.7	0.1
34	0.2	0.9	0.9	0.7	0.8	0.1
35	0.2	0.9	0.9	0.7	0.7	0.1
36	0.2	0.8	0.9	0.7	0.7	0.15
37	0.2	0.8	0.9	0.6	0.7	0.15
38	0.2	0.9	0.8	0.7	0.7	0.15
39	0.2	0.9	0.8	0.7	0.6	0.15
40	0.2	0.9	0.9	0.8	0.7	0.15
41	0.2	0.9	0.9	0.7	0.8	0.15
42	0.2	0.9	0.9	0.7	0.7	0.15
43	0.3	0.8	0.9	0.7	0.7	0.05
44	0.3	0.8	0.9	0.6	0.7	0.05
45	0.3	0.9	0.8	0.7	0.7	0.05
46	0.3	0.9	0.8	0.7	0.6	0.05
47	0.3	0.9	0.9	0.8	0.7	0.05
48	0.3	0.9	0.9	0.7	0.8	0.05
49	0.3	0.9	0.9	0.7	0.7	0.05
50	0.3	0.8	0.9	0.7	0.7	0.1
51	0.3	0.8	0.9	0.6	0.7	0.1
52	0.3	0.9	0.8	0.7	0.7	0.1
53	0.3	0.9	0.8	0.7	0.6	0.1

No.	π	Se_A	Sp_A	Se_B	Sp_B	ω
54	0.3	0.9	0.9	0.8	0.7	0.1
55	0.3	0.9	0.9	0.7	0.8	0.1
56	0.3	0.9	0.9	0.7	0.7	0.1
57	0.3	0.8	0.9	0.7	0.7	0.15
58	0.3	0.8	0.9	0.6	0.7	0.15
59	0.3	0.9	0.8	0.7	0.7	0.15
60	0.3	0.9	0.8	0.7	0.6	0.15
61	0.3	0.9	0.9	0.8	0.7	0.15
62	0.3	0.9	0.9	0.7	0.8	0.15
63	0.3	0.9	0.9	0.7	0.7	0.15

Supplemental Table 4: Results for the absolute difference between the adjusted and the initial sample sizes of discordant cases (minimum, median, maximum), stratified by the prevalence (prev) and the difference between true and initially assumed overall success rate (ω) [**bold** values represent the number of subjects that can be saved compared to the initially planned sample size]

**Minimal discordant rate**		$n_{disc}^{adapt} - n_{disc}^{init}$		
		**Minimum**	**Median**	**Maximum**
**prev=0.1**	$\omega = 0.05$	**-11.8**	**-4.2**	5.6
	$\omega = 0.10$	**-20.1**	**-7.9**	13.3
	$\omega = 0.15$	**-28.7**	**-11.3**	19.0
**prev=0.2**	$\omega = 0.05$	**-3.4**	7.6	25.1
	$\omega = 0.10$	**-6.2**	16.3	50.1
	$\omega = 0.15$	**-7.1**	27.1	77.3
**prev=0.3**	$\omega = 0.05$	3.6	19.1	39.8
	$\omega = 0.10$	10.7	42.3	83.3
	$\omega = 0.15$	18.2	63.4	124.3

Supplemental Table 5: Results for the absolute difference between the adjusted and the initial total sample sizes (minimum, median, maximum), stratified by the prevalence (prev) and the difference between true and initially assumed overall success rate (ω) [**bold** values represent number of subjects that can be saved compared to the initially planned sample size]

**Minimal discordant rate**		$n^{adapt} - n^{init}$		
		**Minimum**	**Median**	**Maximum**
**prev=0.1**	$\omega = 0.05$	**-62.7**	**-31.7**	50.7
	$\omega = 0.10$	**-105.2**	**-59.3**	121.8
	$\omega = 0.15$	**-151.8**	**-84.1**	172.1
**prev=0.2**	$\omega = 0.05$	**-19.4**	56.7	208.8
	$\omega = 0.10$	**-35.2**	122.1	417.5
	$\omega = 0.15$	**-40.1**	202.7	643.9
**prev=0.3**	$\omega = 0.05$	19.8	143.1	307.0
	$\omega = 0.10$	63.4	316.6	640.0
	$\omega = 0.15$	107.1	475.5	956.1

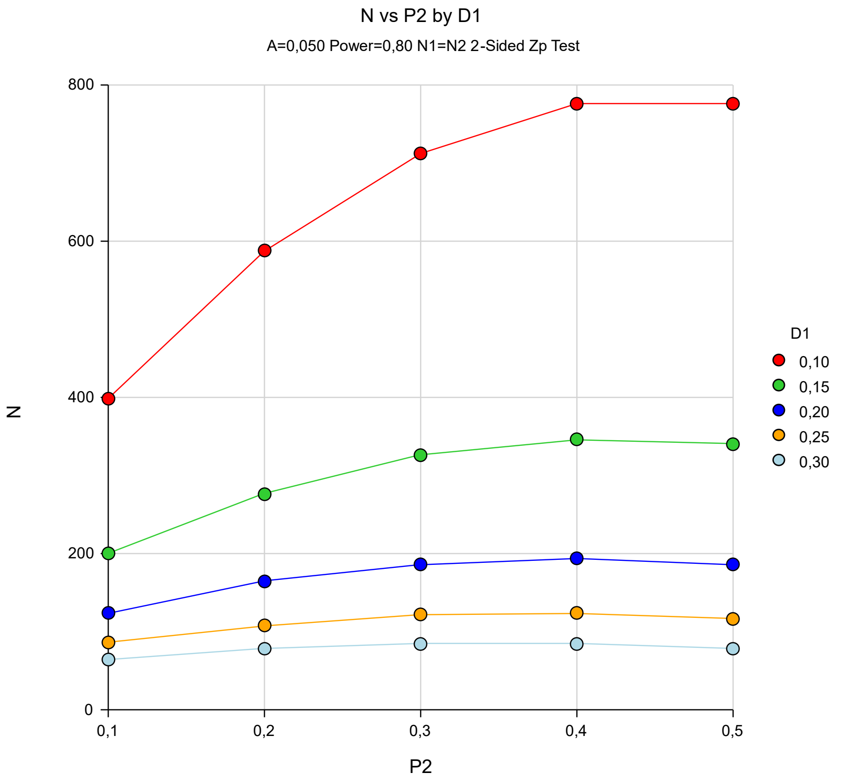

Supplemental Figure 1: Sample size calculation for the test of two proportions using the Z-test with Pooled Variance (using PASS 16.0.3): single success rate (P2) vs. total sample size (N), stratified by the absolute delta (D1), i.e., the absolute difference between the single success rates

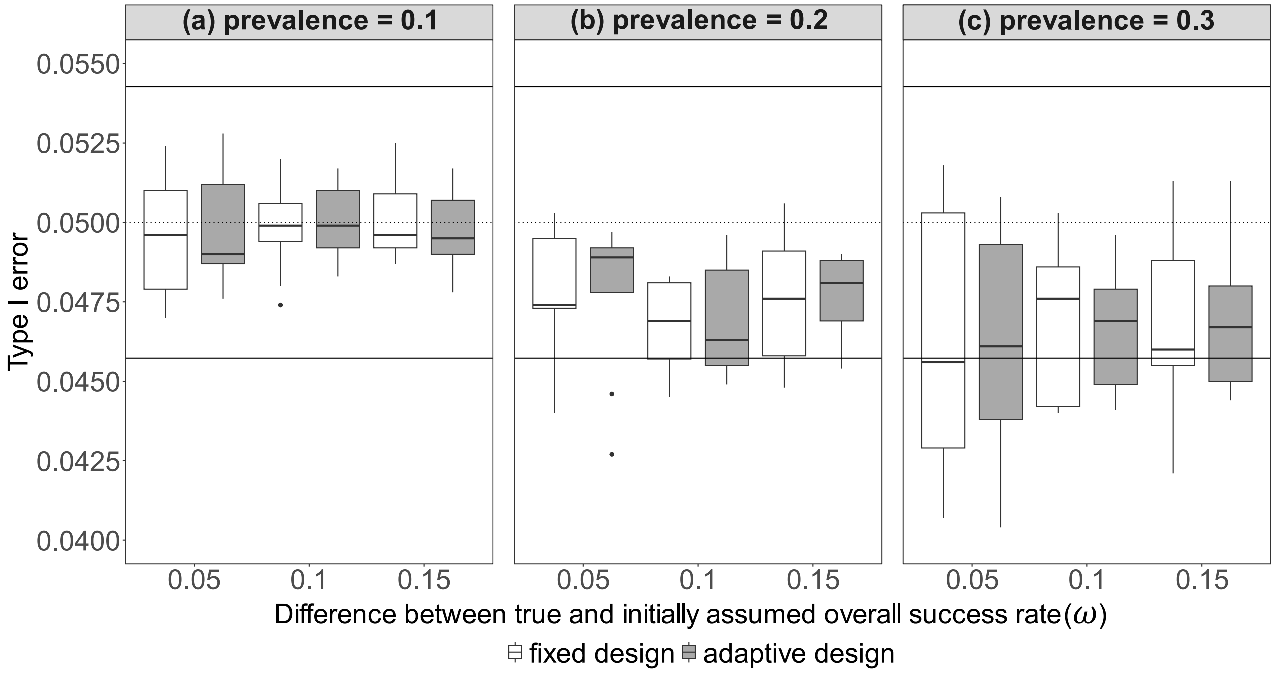

Supplemental Figure 2: Results for the empirical type I error vs. the difference between true and initially assumed overall success rate for the 81 scenarios using mean discordant rate, stratified by the prevalence. The empirical type I error rates for the fixed design and the adaptive design containing a re-estimation of the overall success rate are compared to each other. The black dotted line marks the desired theoretical type I error rate of 5% and the black solid lines mark the respective 95% prediction interval based on the Monte Carlo standard error in the simulation.

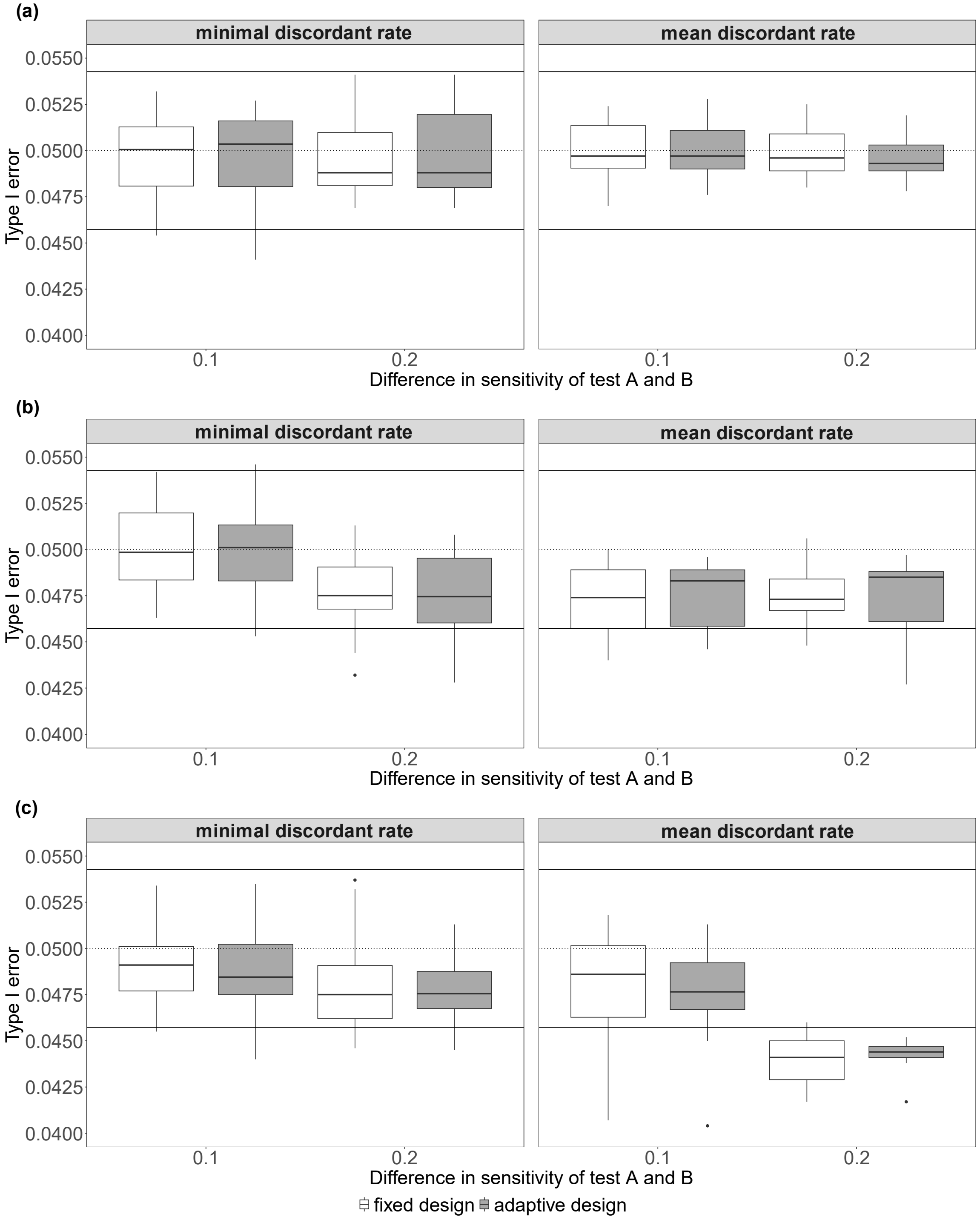

Supplemental Figure 3: Results for the empirical type I error vs. the difference in sensitivity of test A and B for the 225 scenarios, stratified by the discordant rate (figures on the left: minimal discordant rate, figures on the right: mean discordant rate) and the prevalence (Figures (a): prevalence=0.1, Figures (b): prevalence=0.2 and Figures (c): prevalence=0.3). The empirical type I error rates for the fixed design and the adaptive design containing a re-estimation of the overall success rate are compared to each other. The black dotted line marks the desired theoretical type I error rate of 5% and the black solid lines mark the respective 95% prediction interval based on the Monte Carlo standard error in the simulation.

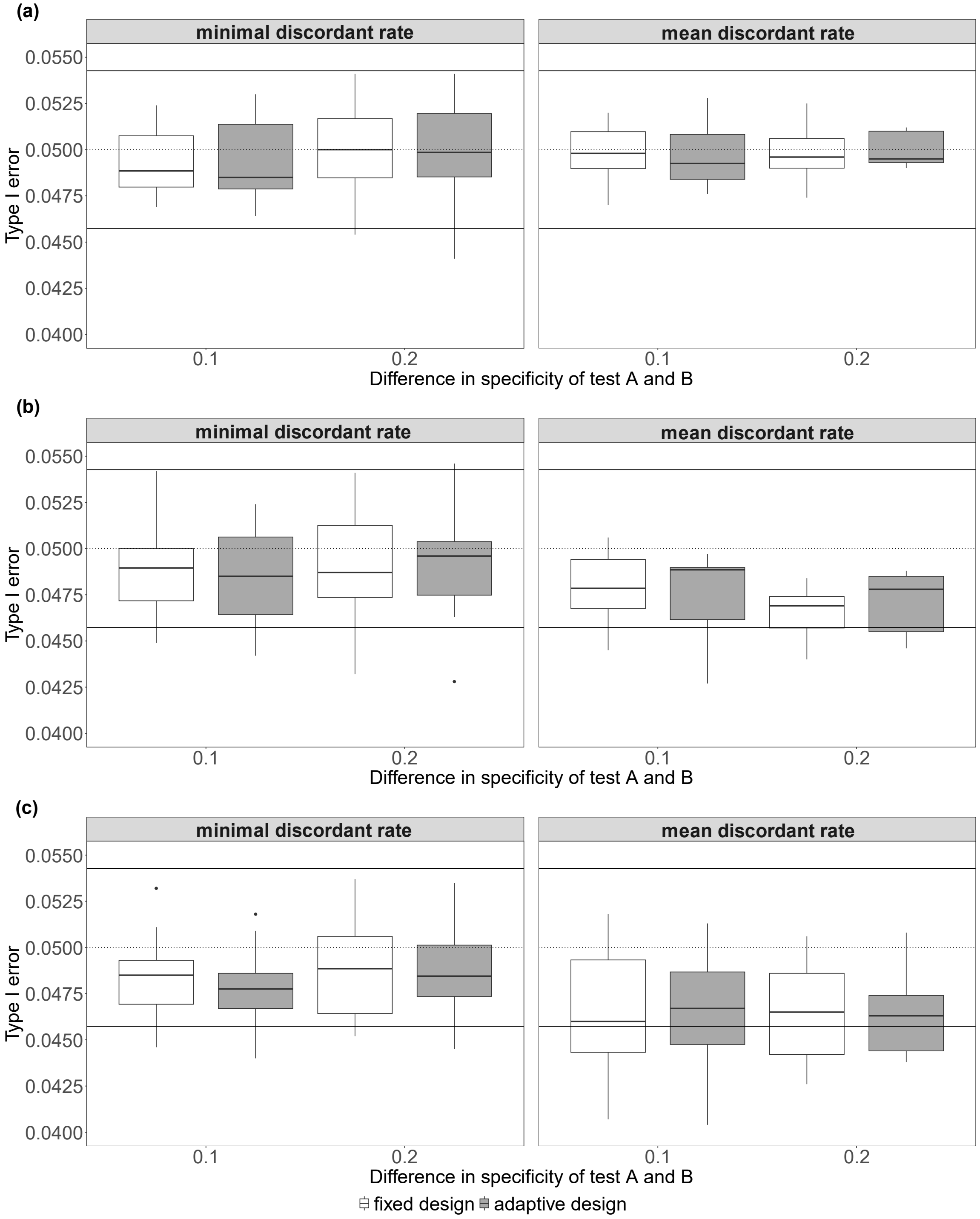

Supplemental Figure 4: Results for the empirical type I error vs. the difference in specificity of test A and B for the 225 scenarios, stratified by the discordant rate (figures on the left: minimal discordant rate, figures on the right: mean discordant rate) and the prevalence (Figures (a): prevalence=0.1, Figures (b): prevalence=0.2 and Figures (c): prevalence=0.3). The empirical type I error rates for the fixed design and the adaptive design containing a re-estimation of the overall success rate are compared to each other. The black dotted line marks the desired theoretical type I error rate of 5% and the black solid lines mark the respective 95% prediction interval based on the Monte Carlo standard error in the simulation.

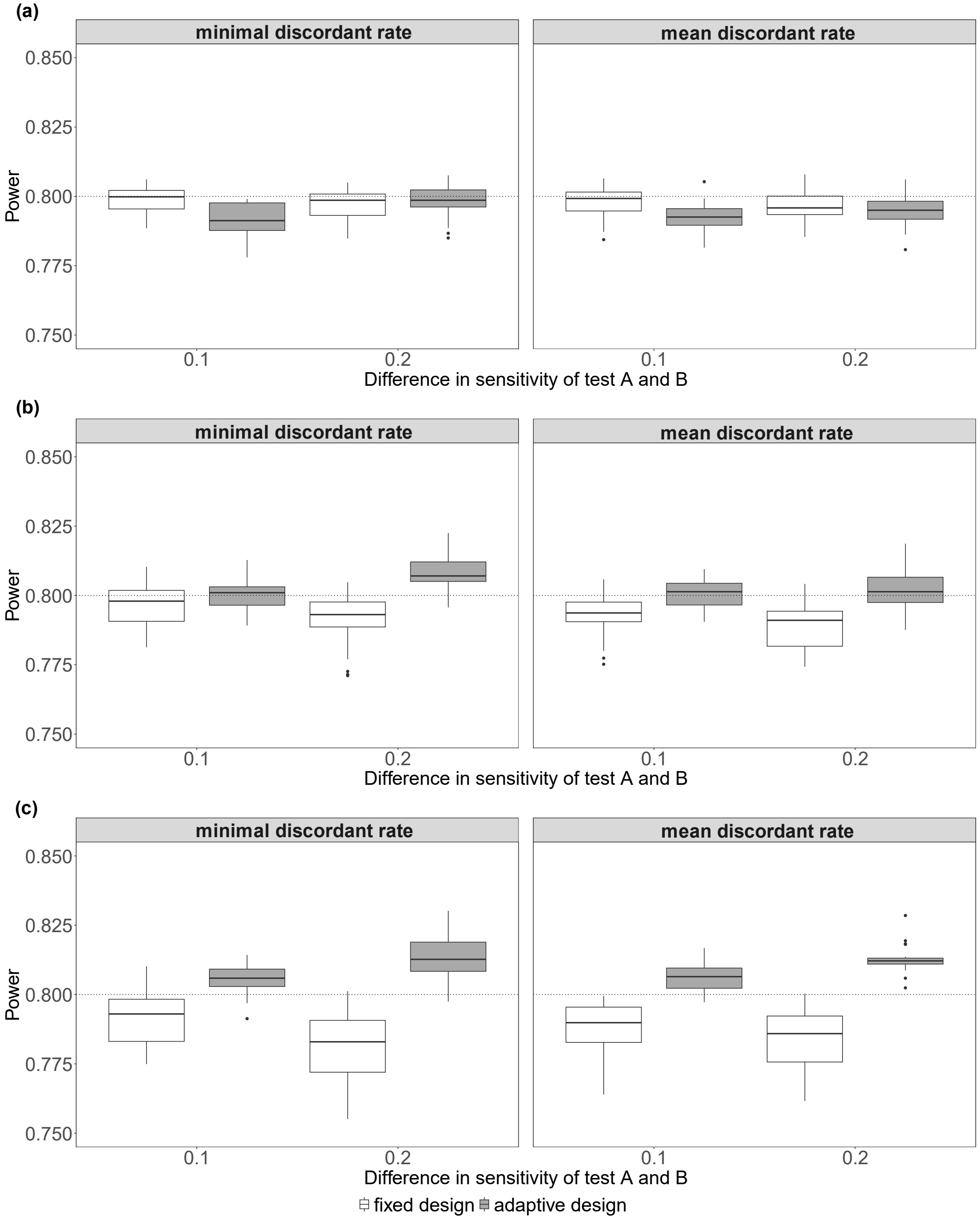

Supplemental Figure 5: Results for the empirical power vs. the difference in sensitivity of test A and B for the 288 scenarios, stratified by the discordant rate (figures on the left: minimal discordant rate, figures on the right: mean discordant rate) and the prevalence (Figures (a): prevalence=0.1, Figures (b): prevalence=0.2 and Figures (c): prevalence=0.3). The empirical power in the fixed design is compared to the adaptive design containing a re-estimation of the overall success rate. The black dotted line marks the desired theoretical power of 80%.

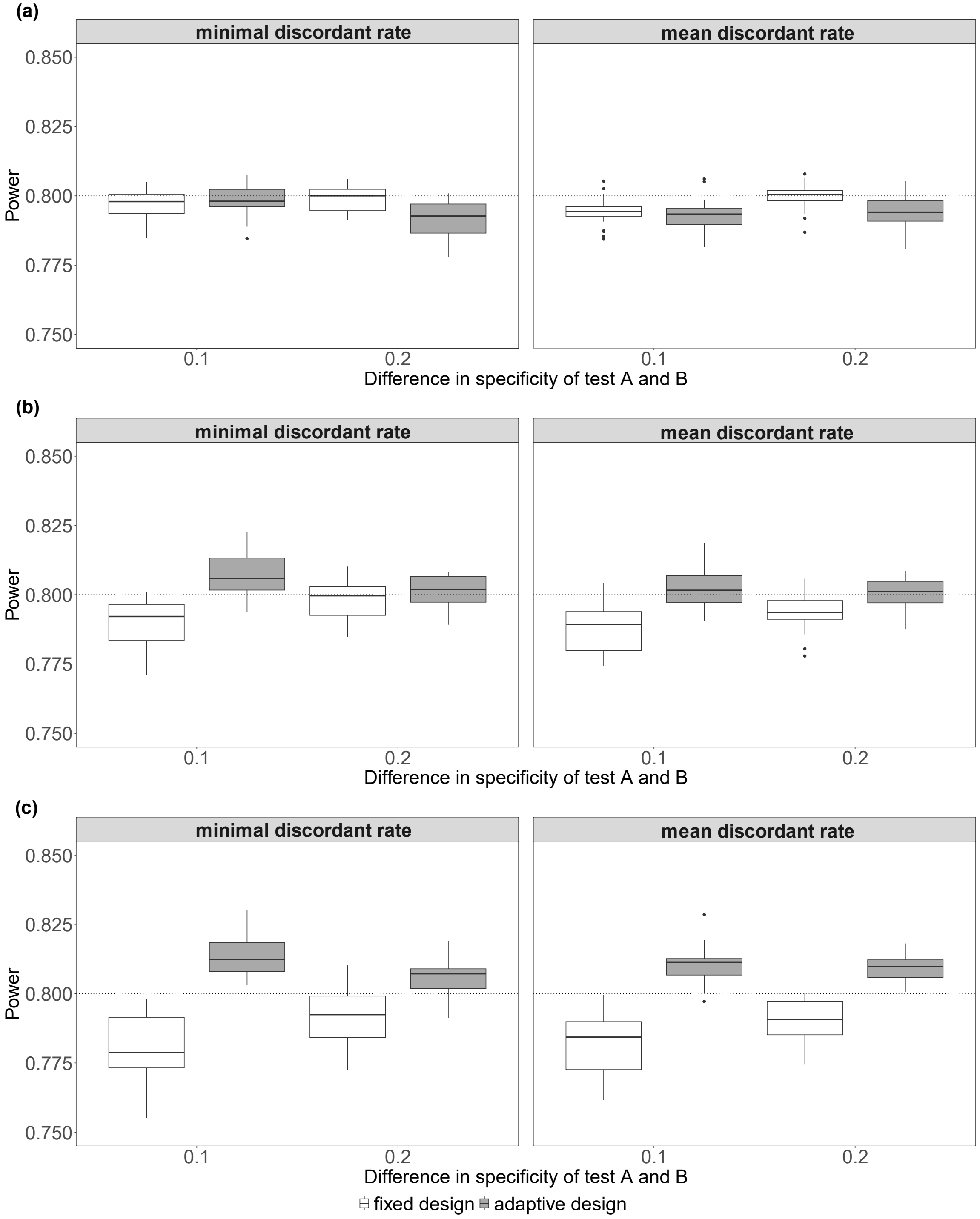

Supplemental Figure 6: Results for the empirical power vs. the difference in specificity of test A and B for the 288 scenarios, stratified by the discordant rate (figures on the left: minimal discordant rate, figures on the right: mean discordant rate) and the prevalence (Figures (a): prevalence=0.1, Figures (b): prevalence=0.2 and Figures (c): prevalence=0.3). The empirical power in the fixed design is compared to the adaptive design containing a re-estimation of the overall success rate. The black dotted line marks the desired theoretical power of 80%.

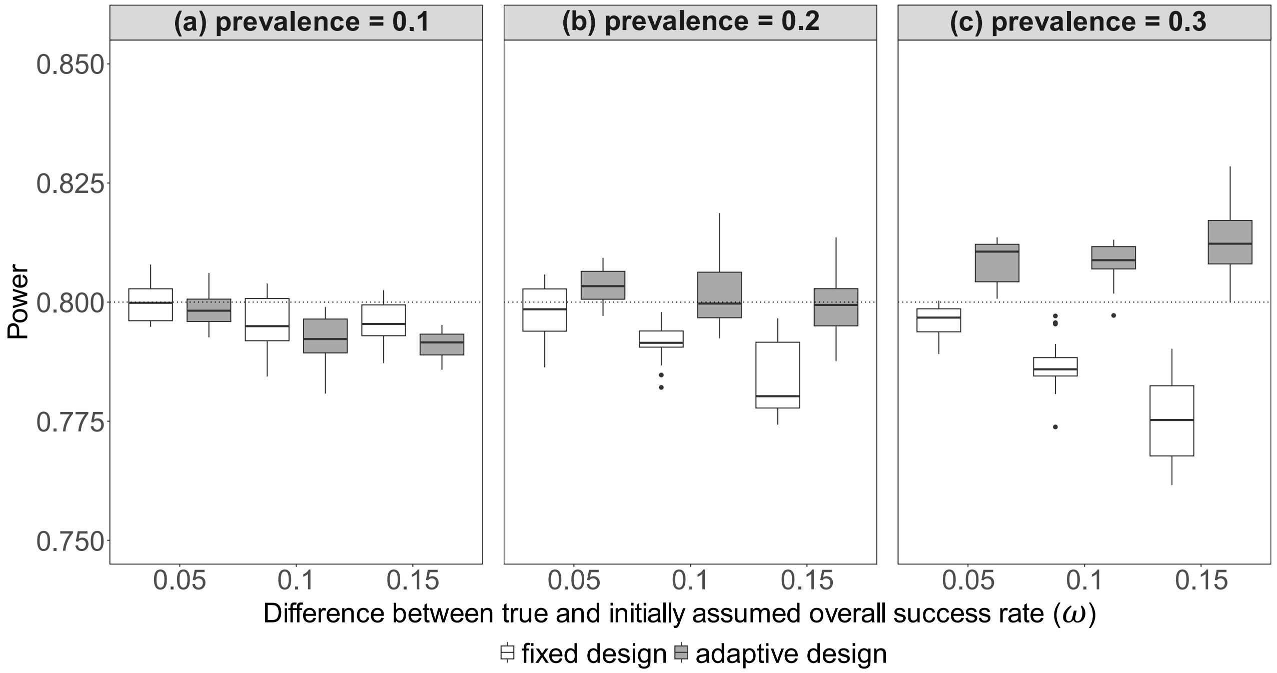

Supplemental Figure 7: Results for the empirical power vs. the difference between true and initially assumed overall success rate for the 144 scenarios using mean discordant rate, stratified by the prevalence. The empirical power in the fixed design is compared to the adaptive design containing a re-estimation of the overall success rate. The black dotted line marks the desired theoretical power of 80%.

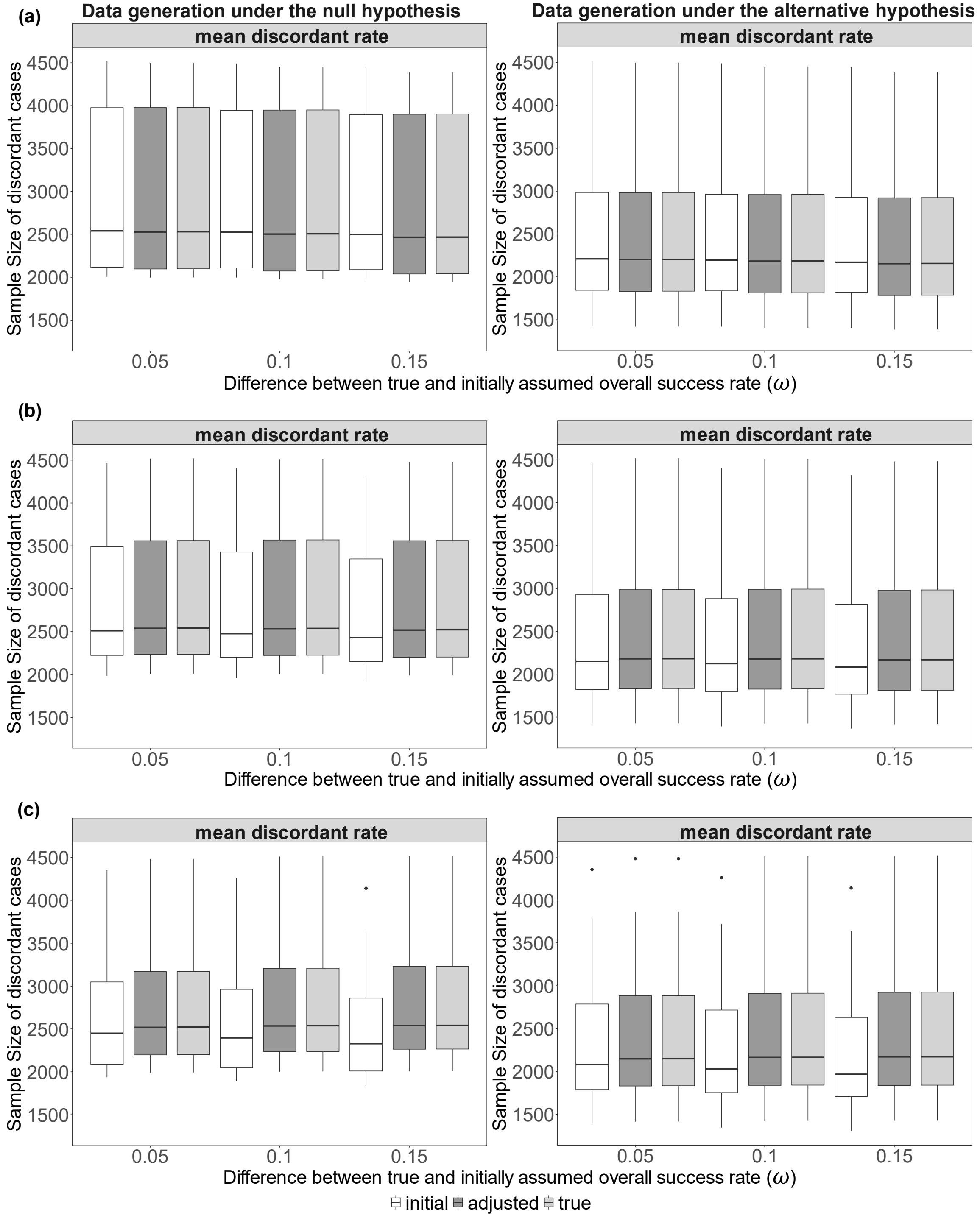

Supplemental Figure 8: Results of the calculated initial, adjusted and true sample sizes of discordant cases vs. the difference between true and initially assumed overall success rate using mean discordant rate, stratified by the data generation method (figures on the left: data generated under the null hypothesis, figures on the right: data generated under the alternative hypothesis) and the prevalence (Figures (a): prevalence=0.1, Figures (b): prevalence=0.2 and Figures (c): prevalence=0.3).

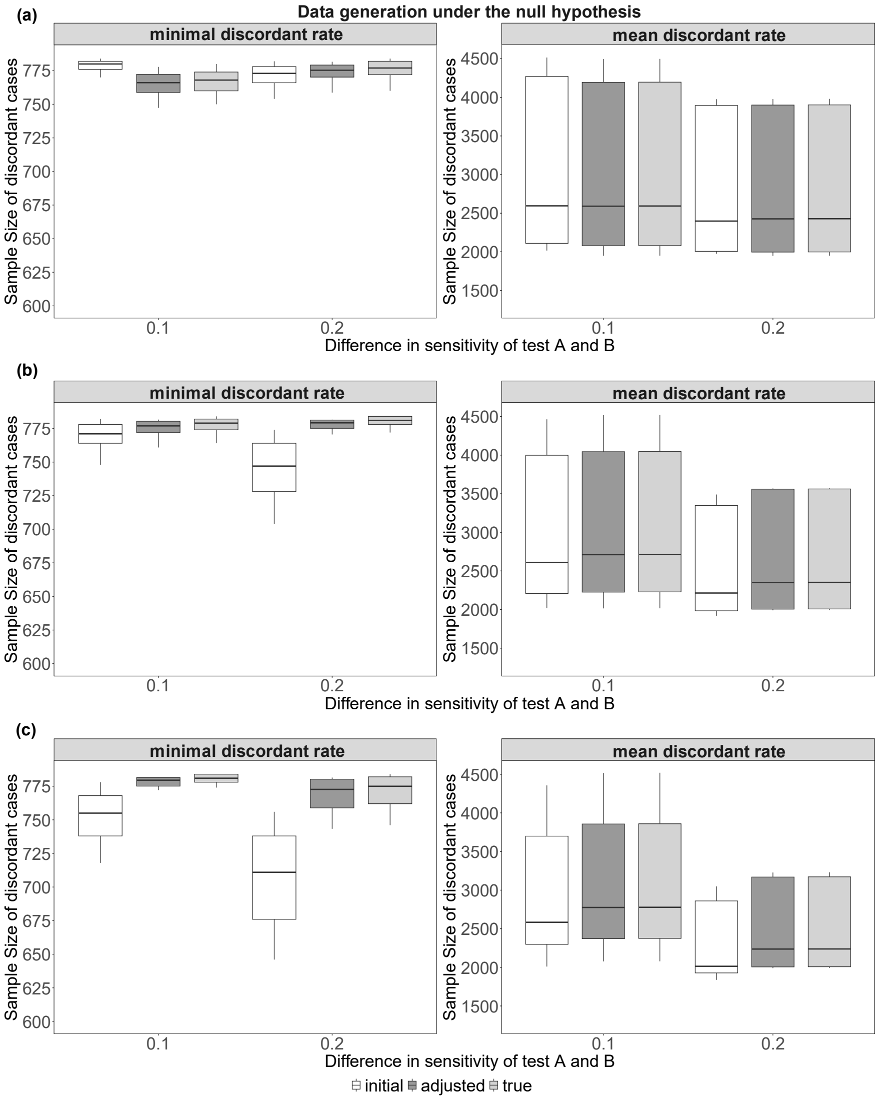

Supplemental Figure 9: Results of the calculated initial, adjusted and true sample sizes of discordant cases vs. the difference in sensitivity of test A and B for the 225 scenarios, stratified by the discordant rate (figures on the left: minimal discordant rate, figures on the right: mean discordant rate) and the prevalence (Figures (a): prevalence=0.1, Figures (b): prevalence=0.2 and Figures (c): prevalence=0.3).

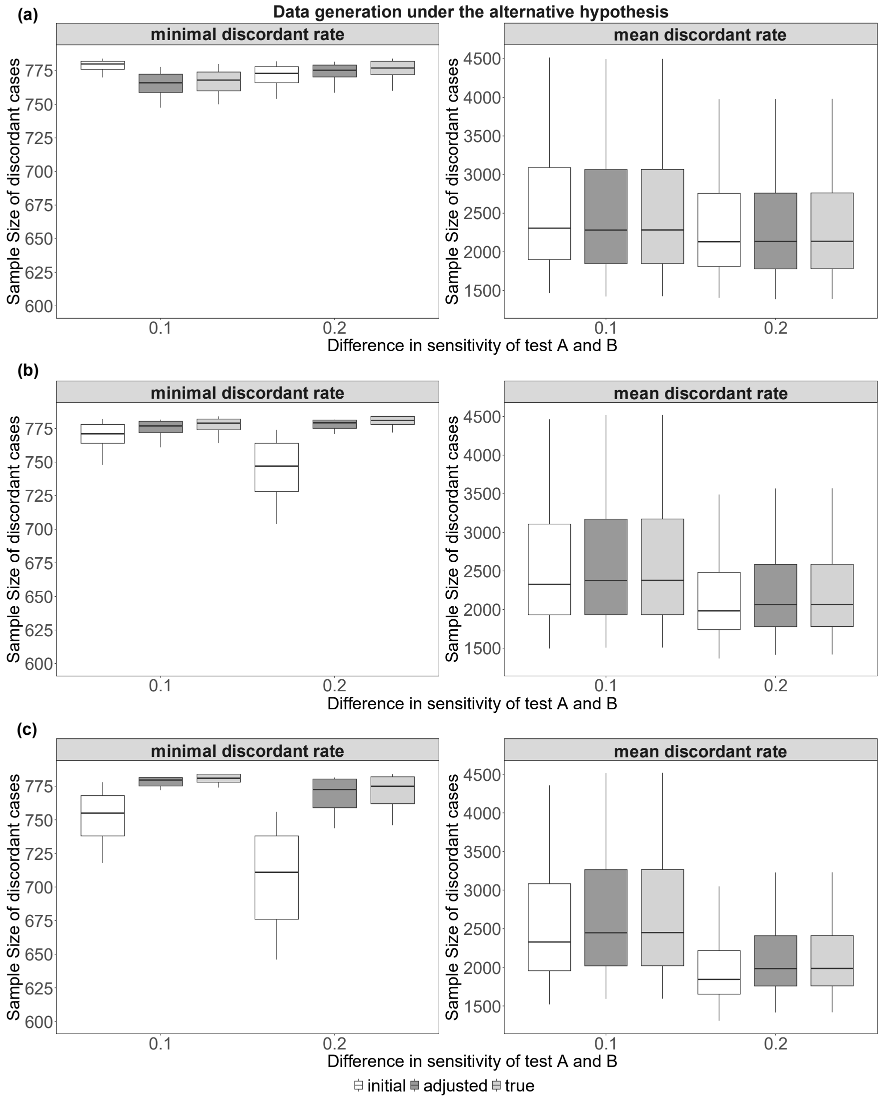

Supplemental Figure 10: Results of the calculated initial, adjusted and true sample sizes of discordant cases vs. the difference in sensitivity of test A and B for the 288 scenarios, stratified by the discordant rate (figures on the left: minimal discordant rate, figures on the right: mean discordant rate) and the prevalence (Figures (a): prevalence=0.1, Figures (b): prevalence=0.2 and Figures (c): prevalence=0.3).

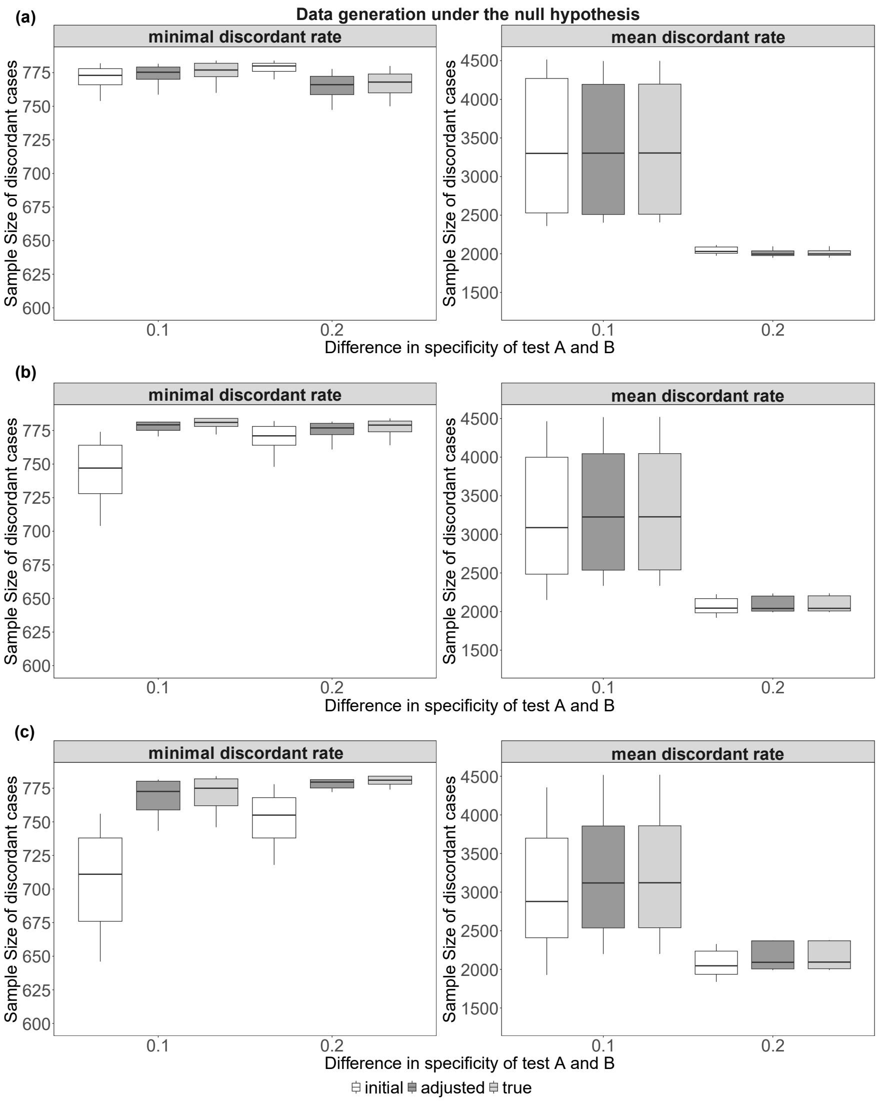

Supplemental Figure 11: Results of the calculated initial, adjusted and true sample sizes of discordant cases vs. the difference in specificity of test A and B for the 225 scenarios, stratified by the discordant rate (figures on the left: minimal discordant rate, figures on the right: mean discordant rate) and the prevalence (Figures (a): prevalence=0.1, Figures (b): prevalence=0.2 and Figures (c): prevalence=0.3).

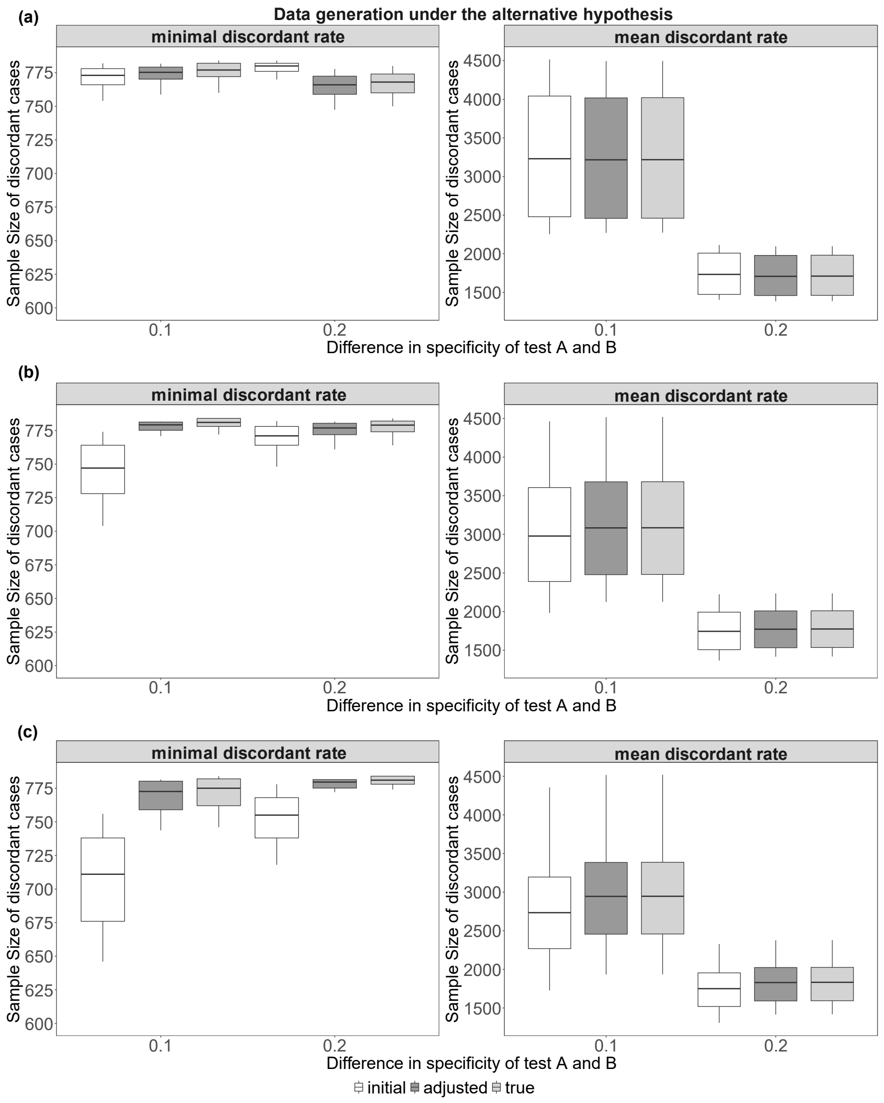

Supplemental Figure 12: Results of the calculated initial, adjusted and true sample sizes of discordant cases vs. the difference in specificity of test A and B for the 288 scenarios, stratified by the discordant rate (figures on the left: minimal discordant rate, figures on the right: mean discordant rate) and the prevalence (Figures (a): prevalence=0.1, Figures (b): prevalence=0.2 and Figures (c): prevalence=0.3).s

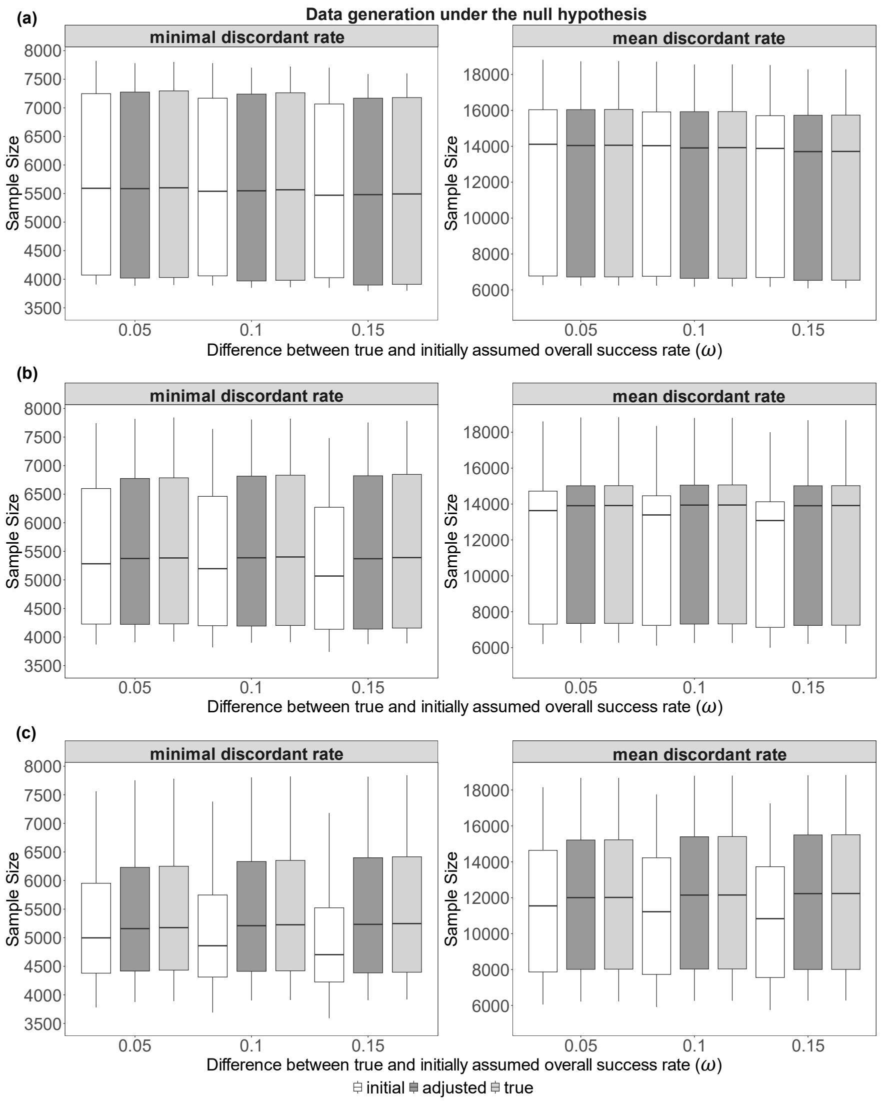

Supplemental Figure 13: Results of the calculated initial, adjusted and true total sample sizes vs. the difference between true and initially assumed overall success rate for the 225 scenarios, stratified by the discordant rate (figures on the left: minimal discordant rate, figures on the right: mean discordant rate) and the prevalence (Figures (a): prevalence=0.1, Figures (b): prevalence=0.2 and Figures (c): prevalence=0.3).

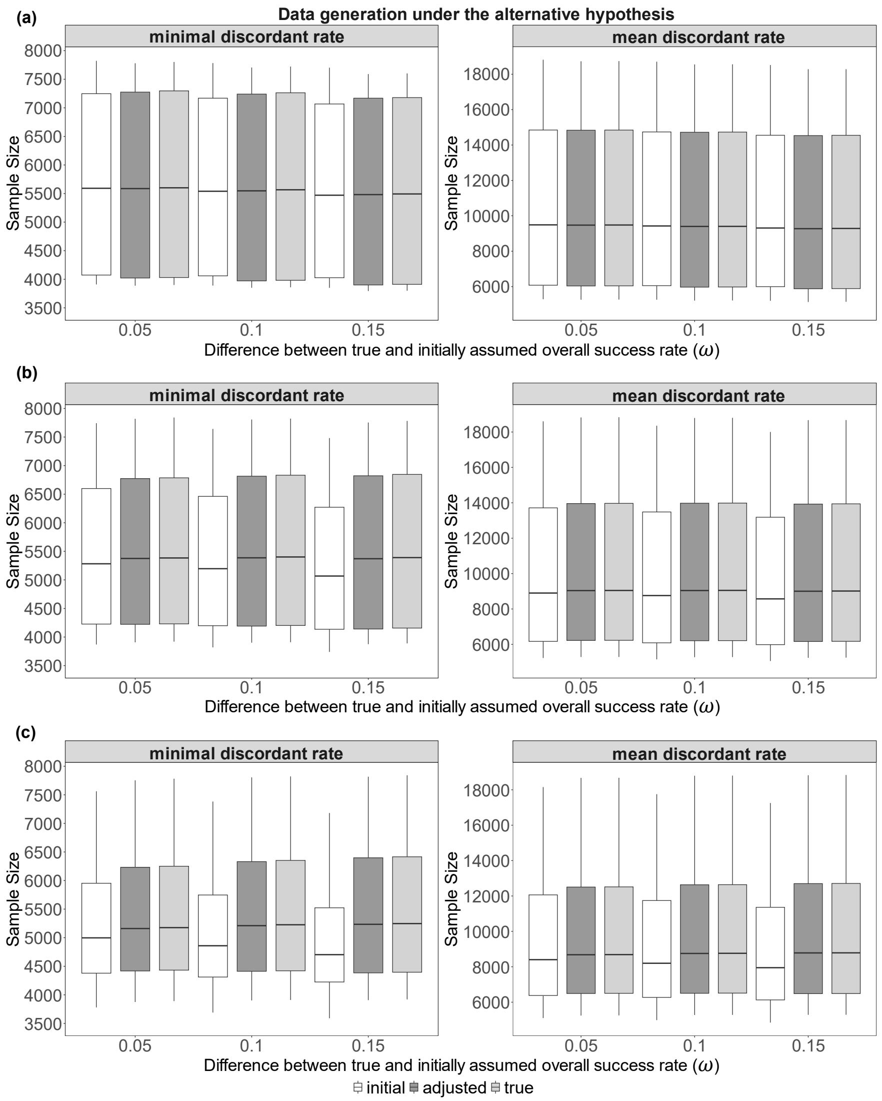

Supplemental Figure 14: Results of the calculated initial, adjusted and true total sample sizes vs. the difference between true and initially assumed overall success rate for the 288 scenarios, stratified by the discordant rate (figures on the left: minimal discordant rate, figures on the right: mean discordant rate) and the prevalence (Figures (a): prevalence=0.1, Figures (b): prevalence=0.2 and Figures (c): prevalence=0.3).

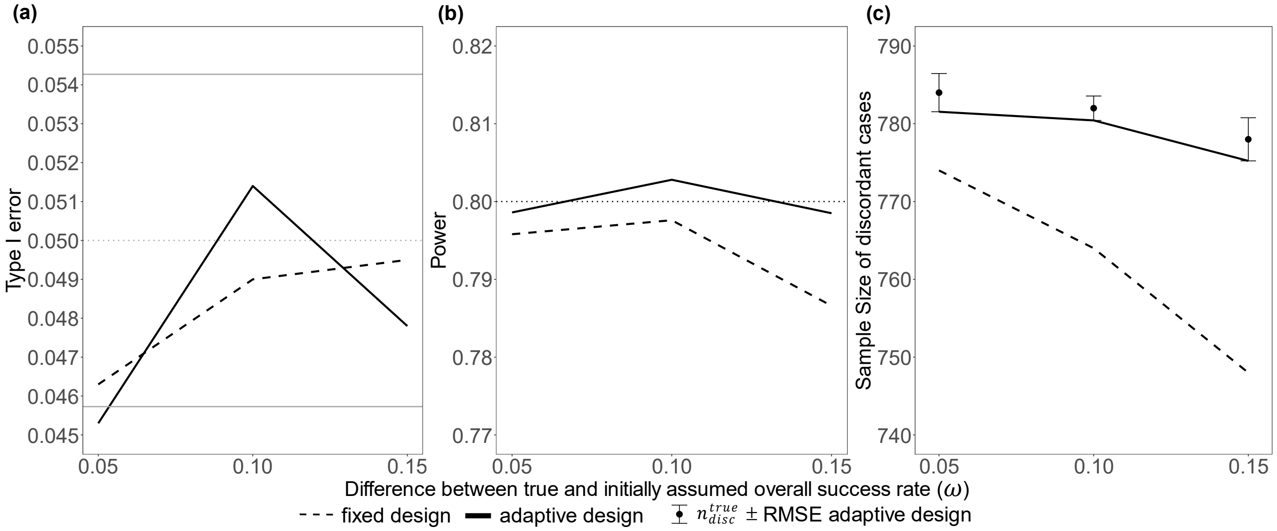

Supplemental Figure 15: Results for the type I error rate (Figure (a)), the power (Figure (b)) and the sample size of discordant cases (Figure (c)) vs. the difference between true and initially assumed overall success rate for the specific scenario (prevalence = 0.2, Se_A= 0.9, Sp_A= 0.8, Se_B= 0.8, Sp_B=0.7) using the minimal discordant rate

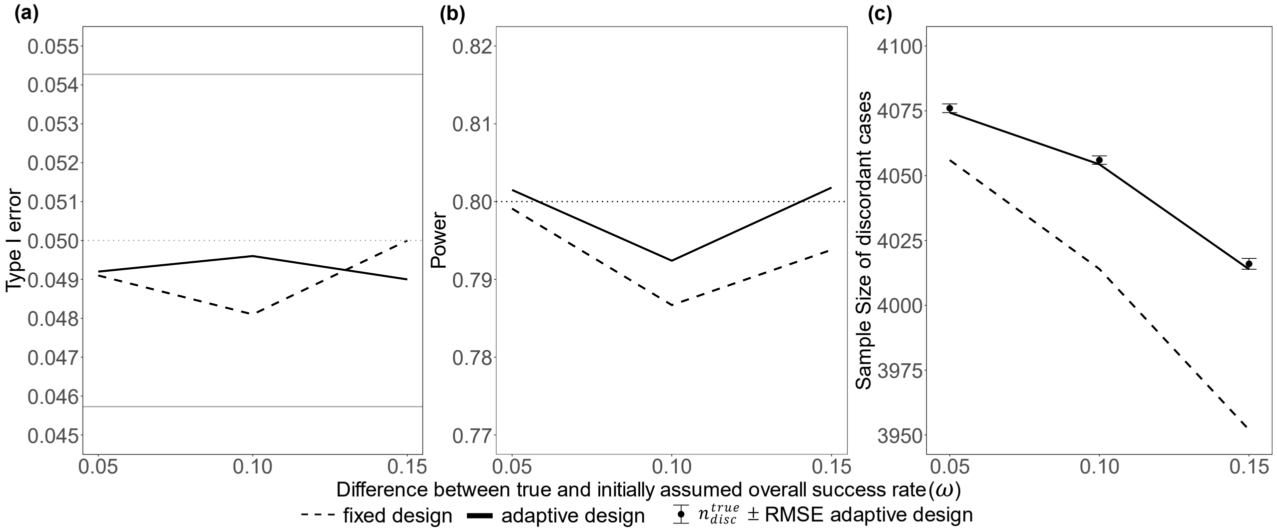

Supplemental Figure 16: Results for the type I error rate (Figure (a)), the power (Figure (b)) and the sample size of discordant cases (Figure (c)) vs. the difference between true and initially assumed overall success rate for the specific scenario (prevalence = 0.2, Se_A= 0.9, Sp_A= 0.8, Se_B= 0.8, Sp_B=0.7) using the mean discordant rate

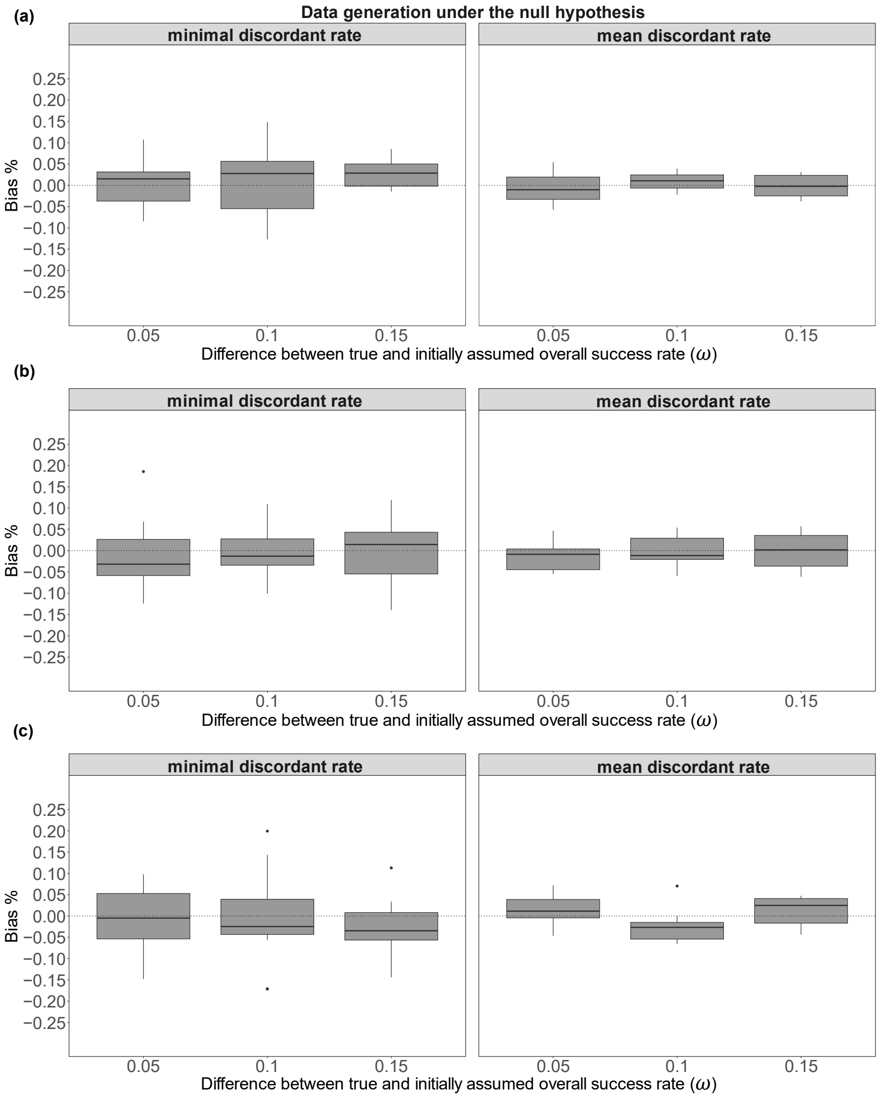

Supplemental Figure 17: Results of the percentage bias regarding the overall success rate vs. the difference between true and initially assumed overall success rate for the 225 scenarios, stratified by the discordant rate (figures on the left: minimal discordant rate, figures on the right: mean discordant rate) and the prevalence (Figures (a): prevalence=0.1, Figures (b): prevalence=0.2 and Figures (c): prevalence=0.3).

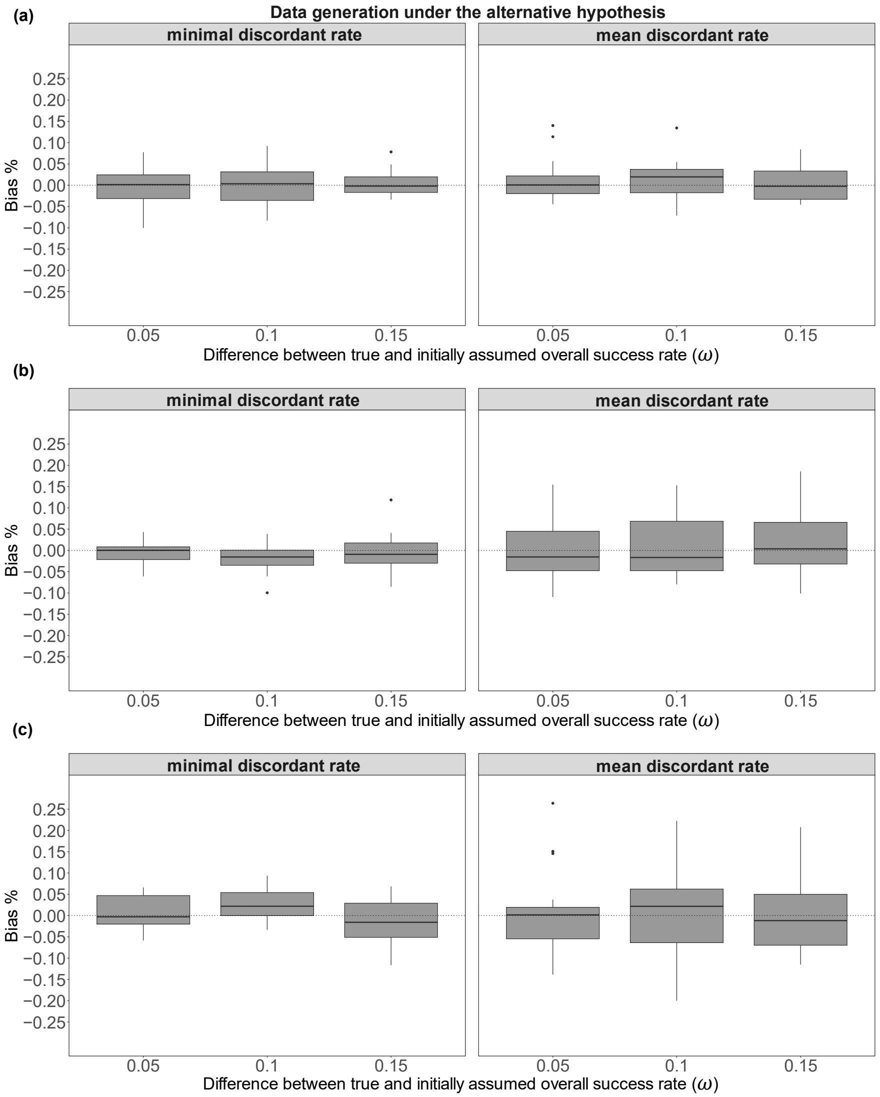

Supplemental Figure 18: Results of the percentage bias regarding the overall success rate vs. the difference between true and initially assumed overall success rate for the 288 scenarios, stratified by the discordant rate (figures on the left: minimal discordant rate, figures on the right: mean discordant rate) and the prevalence (Figures (a): prevalence=0.1, Figures (b): prevalence=0.2 and Figures (c): prevalence=0.3).

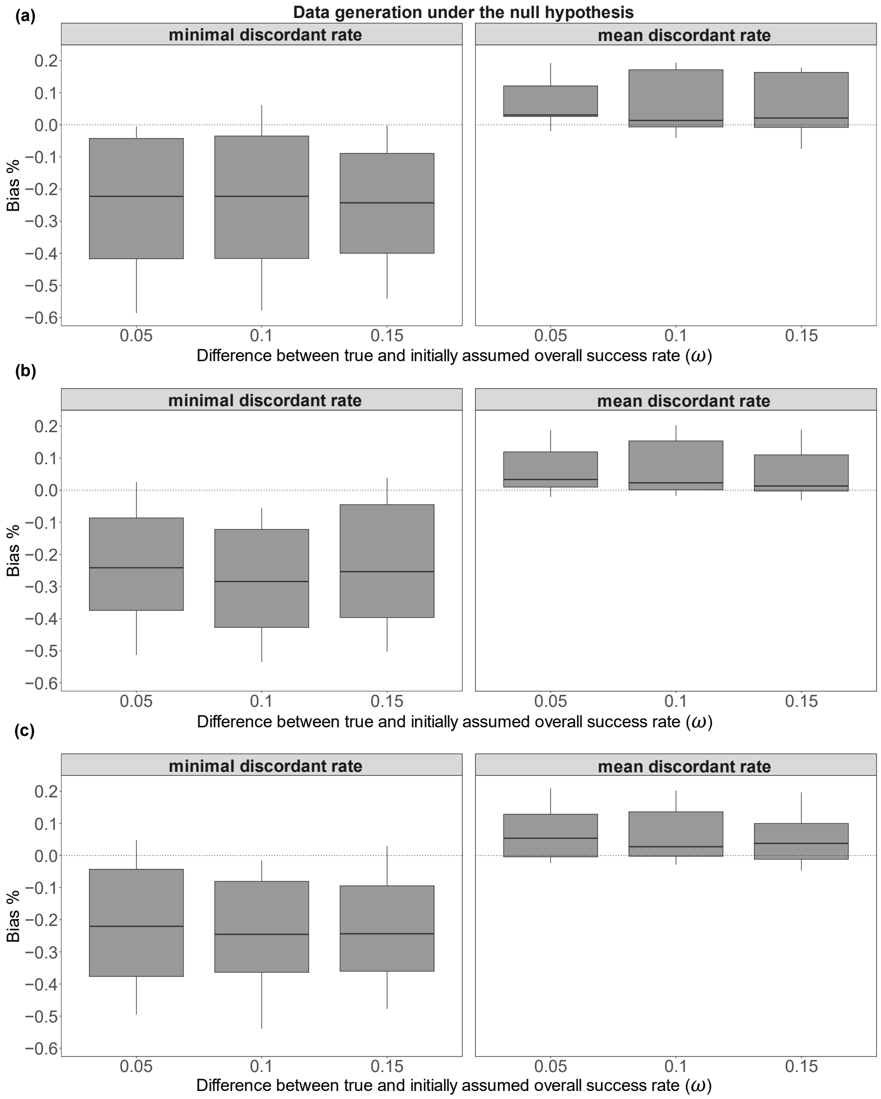

Supplemental Figure 19: Results of the percentage bias regarding the (overall) discordant rate vs. the difference between true and initially assumed overall success rate for the 225 scenarios, stratified by the discordant rate (figures on the left: minimal discordant rate, figures on the right: mean discordant rate) and the prevalence (Figures (a): prevalence=0.1, Figures (b): prevalence=0.2 and Figures (c): prevalence=0.3).

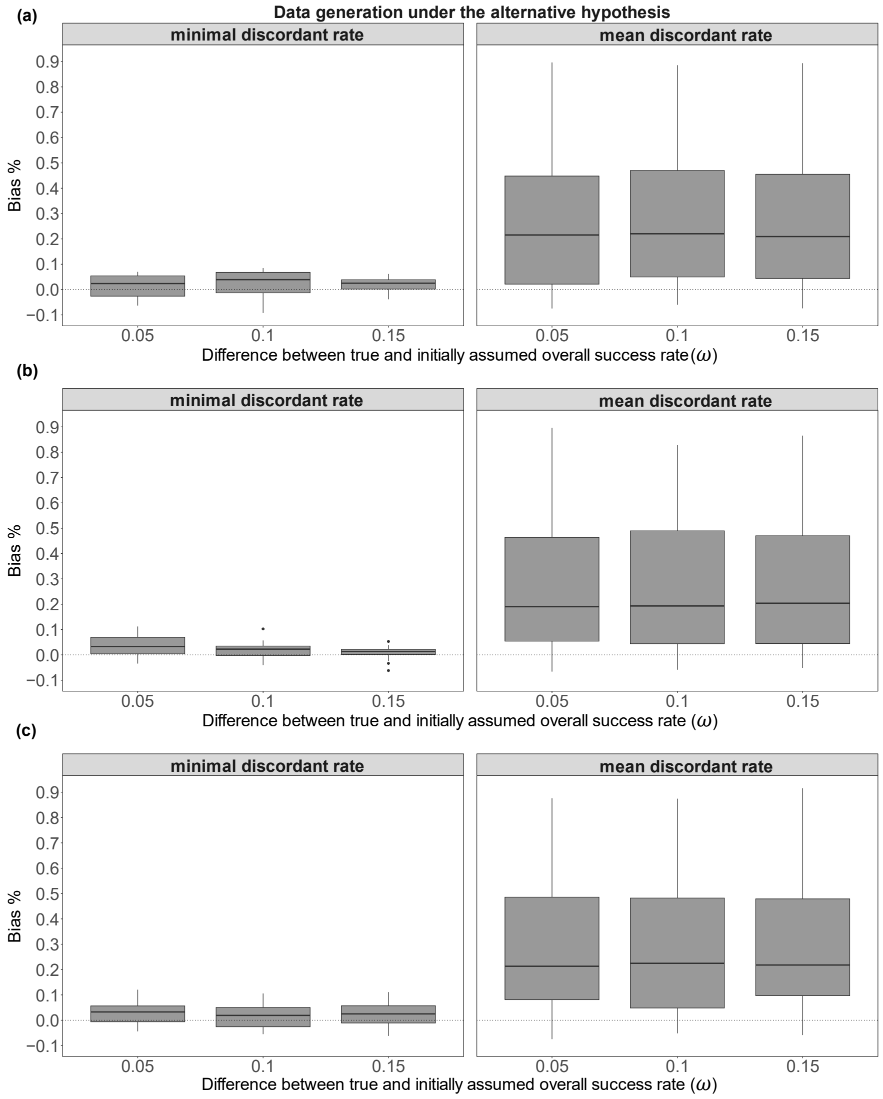

Supplemental Figure 20: Results of the percentage bias regarding the (overall) discordant rate vs. the difference between true and initially assumed overall success rate for the 288 scenarios, stratified by the discordant rate (figures on the left: minimal discordant rate, figures on the right: mean discordant rate) and the prevalence (Figures (a): prevalence=0.1, Figures (b): prevalence=0.2 and Figures (c): prevalence=0.3).

(a)

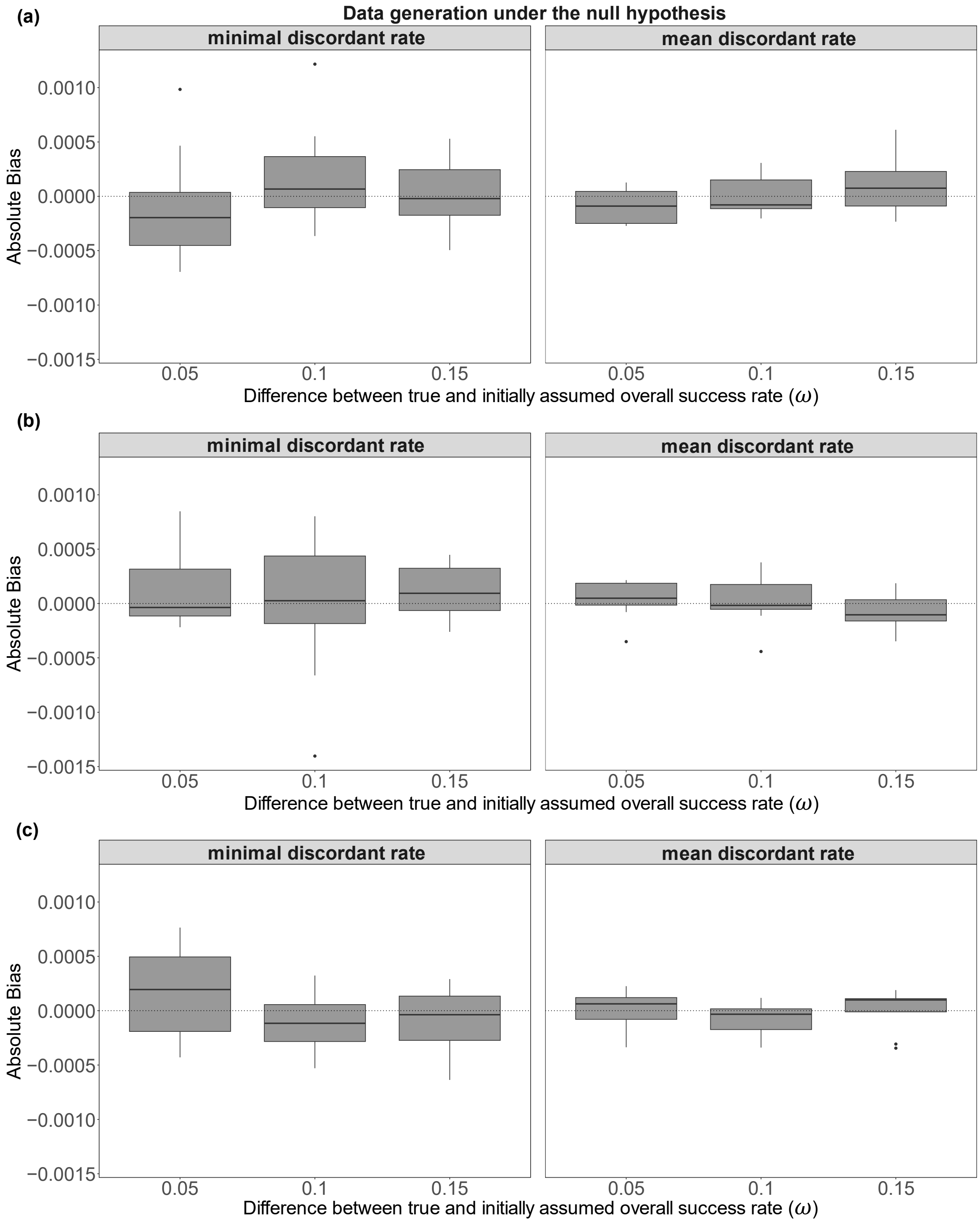

Supplemental Figure 21: Results for the absolute bias regarding the adaptive delta vs. the difference between true and initially assumed overall success rate for the 225 scenarios, stratified by the discordant rate (figures on the left: minimal discordant rate, figures on the right: mean discordant rate) and the prevalence (Figures (a): prevalence=0.1, Figures (b): prevalence=0.2 and Figures (c): prevalence=0.3).

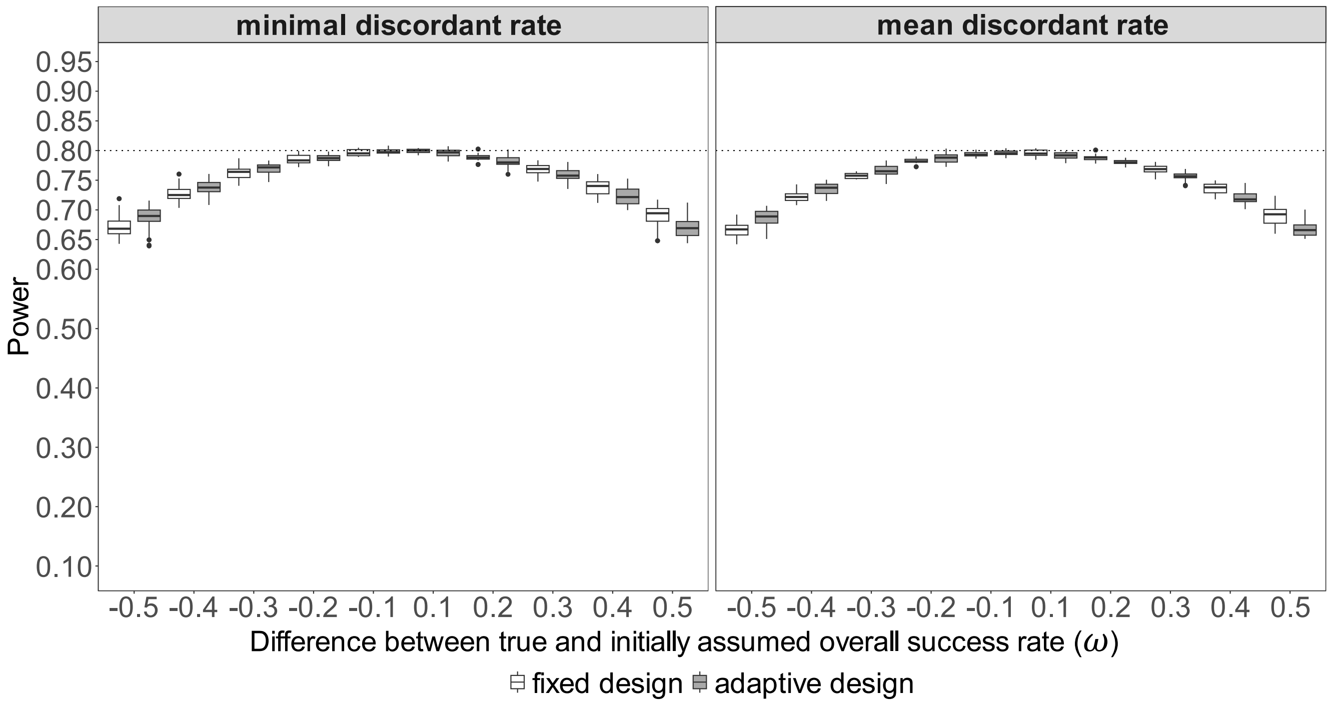

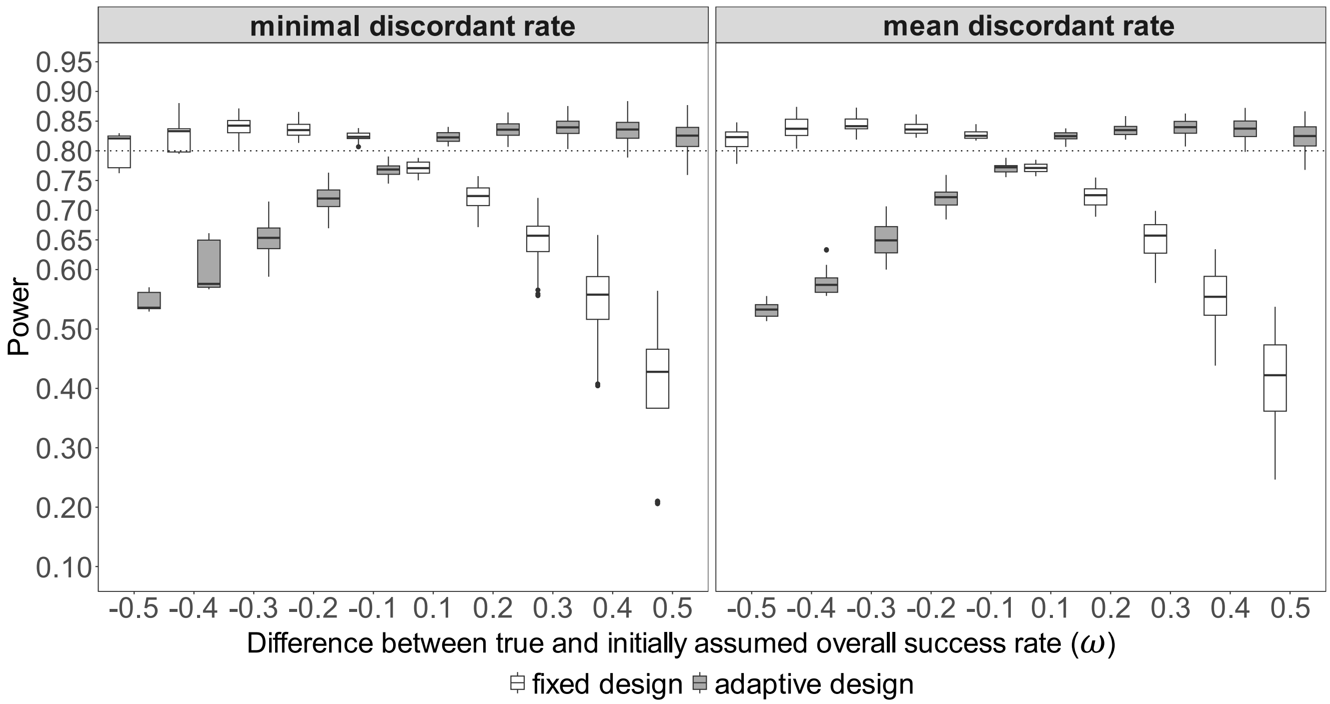

Supplemental Figure 22: Results for the empirical power vs. the difference between true and initially assumed overall success rate for the additional scenarios, stratified by the discordant rate (figures on the left: minimal discordant rate, figures on the right: mean discordant rate) and the prevalence (upper figures: prevalence=0.1, bottom figures: prevalence=0.5). The empirical power in the fixed design is compared to the adaptive design containing a re-estimation of the overall success rate. The black dotted line marks the desired theoretical power of 80%.

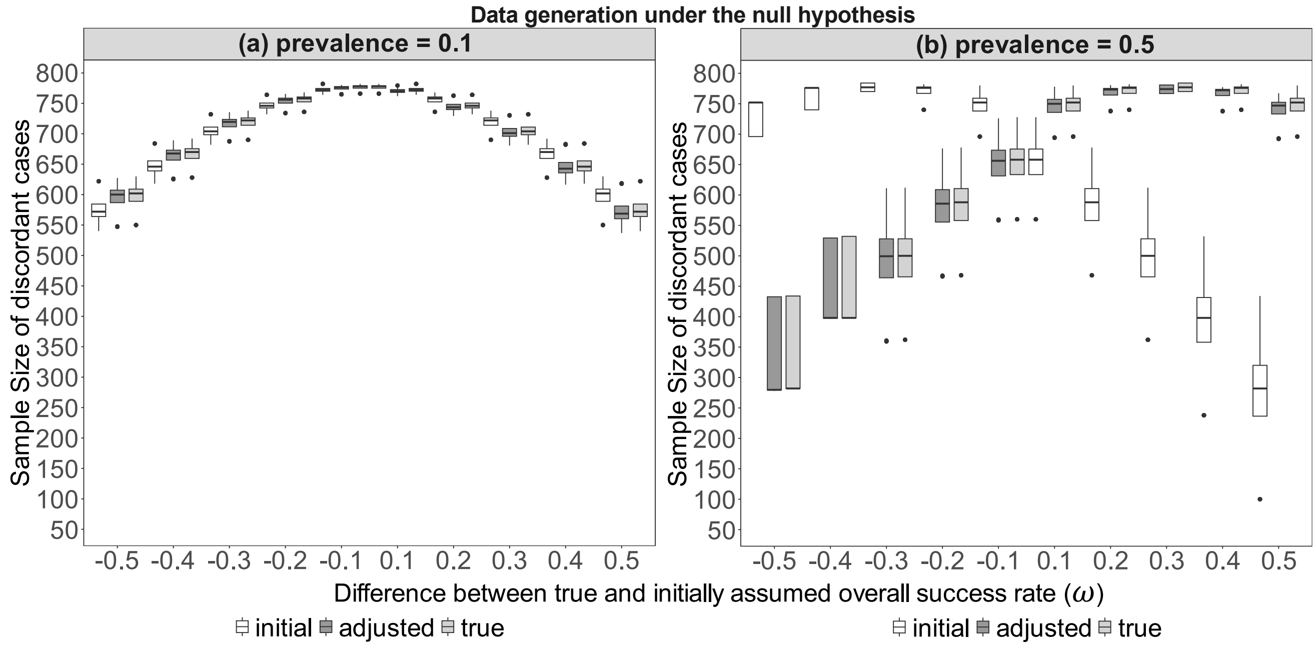

Supplemental Figure 23: Results of the calculated initial, adjusted and true sample sizes of discordant cases vs. the difference between true and initially assumed overall success rate for the additional scenarios, stratified by the prevalence (Figure (a): prevalence=0.1, Figure (b): prevalence=0.5).
The variation in the difference between the true and initially assumed overall success rate on the x-axis results from changes in the initial overall success rate, while the true overall success rate is held constant. This also leads to different initial sample sizes, which in turn helps explain the observed empirical power, as presented in Supplemental Figure 22.
