# Supplementary material for: Sample size recalculation based on the overall success rate in a randomized test-treatment trial with restricting randomization to discordant pairs
Source: BMC Med Res Methodol. 2025 Mar 18;25:74. doi: 10.1186/s12874-024-02410-3 (PMC11921670; doi:10.1186/s12874-024-02410-3)
Supplement: Supplementary file 2 — Supplementary Material 2: Listing of results regarding empirical type I error rates as well as empirical power and R Code. [file 12874_2024_2410_MOESM2_ESM.pdf]

Additional File 2: Supplementary Material

Listing: Empirical Type I error rate

The Listing of the empirical type I error rates for the 225 scenarios (across all 10 000 simulation runs each) is attached in the following electronic file:
Additional_File_2_1_Listing_Type_I_error.csv

Listing: Empirical Power

The Listing of the empirical power for the 288 scenarios (across all 10 000 simulation runs each) is attached in the following electronic file:
Additional_File_2_2_Listing_Power.csv

R code

The simulation study was programmed using RStudio 2021.09.0+351 "Ghost Orchid" Release for Windows (R Software Version 4.2.2). The simulation study was performed with the R packages *foreach*, *parallel*, *doParallel*, *tidyr*, *rlist*, *dplyr*, *randomizr*, *bindata* and *DescTools*.
The R code for the simulation study is attached in the following electronic file:
Additional_File_2_3_R_code.txt
